# Infection and co-infection patterns of community-acquired pneumonia in patients of different ages in China from 2009‒2020: a national surveillance study

**DOI:** 10.1016/S2666-5247(23)00031-9

**Published:** 2023-03-28

**Authors:** Yan-Ning Liu, Yun-Fa Zhang, Qiang Xu, Yan Qiu, Qing-Bin Lu, Tao Wang, Xiao-Ai Zhang, Sheng-Hong Lin, Chen-Long Lv, Bao-Gui Jiang, Hao Li, Zhong-Jie Li, George F. Gao, Wei-Zhong Yang, Simon I. Hay, Li-Ping Wang, Li-Qun Fang, Wei Liu

**Affiliations:** 1 State Key Laboratory of Pathogen and Biosecurity, Beijing Institute of Microbiology and Epidemiology, Beijing, China; 2 Beijing Haidian District Center for Disease Control and Prevention, Beijing, China; 3 Division of Infectious Disease, Key Laboratory of Surveillance and Early-warning on Infectious Disease, Chinese Center for Disease Control and Prevention, Beijing, China; 4 Department of Laboratorial Science and Technology, School of Public Health, Peking University, Beijing, China; 5 Chinese Center for Disease Control and Prevention, Beijing, China; 6 Department of Health Metrics Sciences, School of Medicine, University of Washington; 7 Institute for Health Metrics and Evaluation, University of Washington

## Abstract

**Background:**

Severe community-acquired pneumonia (SCAP) is associated with a substantial number of hospitalisations and deaths worldwide. Infection or co-infection patterns, along with their age dependence and clinical effects are poorly understood. We aimed to explore the causal and epidemiological characteristics by age, to better describe patterns of community-acquired pneumonia (CAP) and their association with severe disease.

**Methods:**

National surveillance of CAP was conducted through a network of hospitals in 30 provinces in China from 2009‒20 inclusive. Patients with CAP were included if they had evidence of acute respiratory tract, had evidence of pneumonia by chest radiography, diagnosis of pneumonia within 24 h of hospital admission, and resided in the study catchment area. For the enrolled patients with CAP, nasopharyngeal and oral swabs were taken and tested for eight viral pathogens; and blood, urine, or expectorated sputum was tested for six bacterial pathogens. Clinical outcomes, including SCAP, were investigated with respect to age and patterns of infections or co-infections by performing binary logistic regression and multivariate analysis.

**Findings:**

Between January, 2009, and December, 2020, 18 807 patients with CAP (3771 [20·05%] with SCAP) were enrolled. For both children (aged ≤5 years) and older adults (aged >60 years), a higher overall rate of viral and bacterial infections, as well as viral–bacterial co-infections were seen in patients with SCAP than in patients with non-SCAP. For adults (aged 18–60 years), however, only a higher rate of bacterial–bacterial co-infection was observed. The most frequent pathogens associated with SCAP were respiratory syncytial virus (RSV; 21·30%) and *Streptococcus pneumoniae* (12·61%) among children, and influenza virus (10·94%) and *Pseudomonas aeruginosa* (15·37%) among older adults. Positive rates of detection of most of the tested pathogens decreased during 2020 compared with the 2009–19 period, except for RSV, *P aeruginosa*, and *Klebsiella pneumoniae*. Multivariate analyses showed SCAP was significantly associated with infection with human adenovirus, human rhinovirus, *K pneumoniae*, or co-infection of RSV and *Haemophilus influenzae* or RSV and *Staphylococcus aureus* in children and adolescents (aged <18 years), and significantly associated with infection with *P aeruginosa*, *K pneumoniae*, or *S pneumoniae*, or co-infection with *P aeruginosa* and *K pneumoniae* in adults (aged ≥18 years).

**Interpretation:**

Both prevalence and infection pattern of respiratory pathogens differed between patients with SCAP and patients with non-SCAP in an age- dependent manner. These findings suggest potential advantages to age-related strategies for vaccine schedules, as well as clinical diagnosis, treatment, and therapy.

**Funding:**

China Mega-Project on Infectious Disease Prevention and The National Natural Science Funds of China.

## Introduction

Pneumonia resulted in nearly 2∙5 million deaths worldwide in 2019,^1^ the fourth highest global cause of death.^2^ In addition to high morbidity and mortality,^3^ pneumonia is also one of the most expensive conditions to treat when hospitalisation is required, with an estimated total cost of $6·4 billion in US hospital settings in 2017.^4^ Moreover, pneumonia continues to be the major killer of young children in low-income countries and older people in high-income countries.^5^ Widespread introduction of *Haemophilus influenzae* type b and pneumococcal conjugate vaccines into immunisation programmes has led to speculation about the growing predominance of viruses as causes of pneumonia.^5^ However, bacteria continue to have a predominant role in pneumonia in adults,^5^ and evidence of viral-bacterial co-infection remains common. Emergence of COVID-19 re-emphasised the role of respiratory viruses as causes of severe pneumonia,^6^ and raised the concern that viral–bacterial co-infection could be a substantial complication leading to higher morbidity and adverse prognosis.^7^

In the past decade, few studies were designed to comprehensively assess both bacterial and viral cause, or their co-infection patterns in pneumonia, and were often further complicated by inconsistent case definitions for community-acquired pneumonia (CAP), different diagnostic methods used, and the testing of a small range of target pathogens. Understanding the proportion of patients with CAP with respiratory viral-bacterial co-infection is crucial in the responsible use of antibiotics and reduces negative consequences of overuse.^8^ This knowledge could have an effect on refining empirical antibiotic management guidelines for patients with CAP of different ages.^9^

By performing a retrospective observational analysis on the national surveillance data on CAP over a 12-years period, we aimed to explore the causal and epidemiological characteristics by age, to better describe community patterns of CAP and their association with severe disease, which in turn might help refine evidence-based strategies to reduce morbidity and mortality.

## Methods

### Study design and case definition

A national surveillance project on acute respiratory tract infections^10^ was completed under the leadership of the China Center for Disease Control and Prevention (CDC) in 30 provinces in mainland China (2009‒20). Patients with CAP were included if they met the following criteria: had evidence of acute respiratory tract infections (detailed in appendix 2 pp 1–2), accompanied by evidence consistent with pneumonia as assessed by chest radiography, diagnosis of pneumonia that was obtained within 24 h of hospital admission, and resided in the study catchment area. All radiographical evidence suggestive of pneumonia required independent interpretation of chest radiographs by a certified radiologist—ie, presence of consolidation (a dense or fluffy opacity with or without air bronchograms), other infiltrate (linear and patchy alveolar or interstitial densities), or pleural effusion. Patients were excluded if they had an alternative diagnosis of a respiratory disorder, had an immunodeficient status, had received a solid-organ or haematopoietic stem-cell transplant within previous 90 days, had a residence outside the study catchment area, were referred from other hospitals, or not initially diagnosed in sentinel hospitals. Further definition of severe CAP (SCAP) was made separately for patients younger than 18 years based on the guidelines for the management of common childhood illnesses^11^ and for patients aged 18 years or older^12^ (see full definitions in appendix 2 pp 1–2). The surveillance protocol was reviewed and approved by the ethics review committees of the China CDC (2015–025).^13^ Due to data from patients with CAP being part of ongoing public health surveillance and implemented national surveillance guidelines, the National Health Commission of the People’s Republic of China has decided that a brief verbal consent was only required to be provided by parents or guardians of participants in this study during their enrolment, which was recorded in each questionnaire by their physicians.

### Procedures

Data including demography, timeline of symptom onset, hospital admission and intensive care unit (ICU) admission (if any), clinical manifestations, laboratory testing results, microbiological test results, chest radiographical findings, and clinical outcomes were collected by reviewing medical records, and entered into a standardised database by trained clinicians. The collected samples included nasopharyngeal and oral swabs to test for viral pathogens and blood, urine, or expectorated sputum to test for bacterial pathogens. Bacterial cultures from blood and expectorated sputum were used to test for *Streptococcus pneumoniae*, *Staphylococcus aureus*, *Klebsiella pneumoniae*, *Pseudomonas aeruginosa*, and *Haemophilus influenzae*, and bacterial cultures from urine were used to test for *S pneumoniae* antigen. RT-PCR was performed to test for the presence of influenza virus (IFV), respiratory syncytial virus (RSV), human parainfluenza virus (HPIV), human metapneumovirus (HMPV), seasonal human coronavirus (HCoV), and human rhinovirus (HRV). PCR was performed to test for the presence of human adenovirus (HAdV), human bocavirus (HBoV), and *Mycoplasma pneumoniae*. All tests followed a standard operating protocol developed by the China CDC (appendix 2 pp 1–2).^13^ There was no difference in sample collection or test strategies between patients with SCAP and with non-SCAP. Positive test results were excluded if they were obtained on samples collected on the third day of admission. The three-day limitation was selected to discriminate community-acquired from nosocomial infections. Each positive result was reviewed by at least one infectious disease physician. Co-infection was determined if tests were positive for more than one of the previously mentioned respiratory pathogens.

### Outcomes

The primary outcome was to assess the patterns of infection and co-infection of viral and bacterial pathogens among patients of different ages with SCAP and non-SCAP. The secondary outcome was intensive care unit admission or not for patients with CAP.

### Statistical analysis

Descriptive statistics included frequencies (proportions) for categorical variables, and medians with IQRs for continuous variables. Either χ^2^ test or Fisher’s extract test was used for the inter-group comparison. A binary logistic regression model was used to examine variables that were related to SCAP and ICU admission, with sex, age, season of infection (cold season or warm season based on the climate characteristics of the sentinel city at the date of onset), and infection of pathogens included as explanatory variables. Multivariate analysis was performed by including all variables with p <0·10 from the univariate analysis as covariates. The odds ratios (ORs) and 95% CIs were estimated using maximum likelihood methods. A conditional logistic regression was performed to examine the potential association between death outcome and the positive tests for respiratory pathogens for adults aged 18 years or older (detailed in appendix 2 pp 1–2) All the statistical analysis was performed using R version 4.1.1. p <0·05 was considered statistically significant.

### Role of the funding source

The funders of the study had no role in study design, data collection, data analysis, data interpretation, or writing of the report.

## Results

Of the 18 807 patients with CAP included in this study between January, 2009, and December, 2020, 3771 (20·05%) were defined as having SCAP, 2183 (11·61%) required ICU admission, and 150 (0·80%) died in hospital. Among all 18 807 patients, 13 009 were tested for all eight viral pathogens, 5178 for all six bacterial pathogens, and 3552 for all 14 viral and bacterial pathogens. A higher proportion of SCAP than non-SCAP was seen in males (68·36% vs 62·29%), older adults (aged >60 years; 35·38% vs 19·89%), and inpatients (87·80% vs 74·28%; all p<0·0001). No difference in proportions of SCAP versus non-SCAP was seen between cold and warm seasons (table).

Among the 13 009 patients with CAP tested for all the eight viral pathogens, 4766 (36·64%) had at least one positive detection. The highest rate of viral pathogen detection was seen in children (aged ≤5 years; 50·82% [3390/6671]), reduced to 32·53% (322/990) in adolescents aged 6–17 years, 20·33% (529/2602) in adults aged 18–60 years, and 19·12% (525/2746) in adults older than 60 years. The positive rate did not differ between sexes but was significantly higher in inpatients than outpatients (38·19% [4030/10 553] vs 29·97% [736/2456], p<0·0001) and in cold season than in warm season (39·17% [2881/7356] vs 33·35% [1885/5653], p<0·0001). IFV had the highest positive rate (in 10·74% [1397/13 009] of CAP), followed by RSV (10·50% [1366/13 009]), HRV (6·78% [882/13 009]), HPIV (6·66% [867/13 009]), HAdV (3·83% [498/13 009]), HCoV (2·51% [326/13 009]), HBoV (2·46% [320/13 009]), and HMPV (2·30% [299/13 009]). The rankings for the top three most prevalent viral pathogens were different across age groups with the rank being: (1) RSV, (2) IFV, and (3) HPIV for children; (1) IFV, (2) HRV, and (3) HAdV for adolescents; and (1) IFV, (2) HRV, and (3) HPIV for both adults and older adults (appendix 2 pp 3–4).

Among the 5178 patients with CAP tested for all six bacterial pathogens, 27·08% (1402) had at least one positive detection. The overall positive rate was significantly higher in patients younger than 18 years than patients aged 18 years or older (34·47% [609/1767]vs 23·25% [793/3411]), in males than females (28·68% [953/3323] vs 24·20% [449/1855]), and in inpatients than outpatients (27·60% [1312/4753] vs 21·18% [90/425]); all p<0·010), but did not differ between season of infection. *S. pneumoniae* had the highest positive rate in patients with CAP (7·49% [388/5178]), followed by *K pneumoniae* (6·95% [360/5178]), *P aeruginosa* (5·81% [301/5178]), *M pneumoniae* (4·79% [248/5178]), *H influenzae* (3·36% [174/5178]), and *S aureus* (3·26% [169/5178]; appendix 2 pp 3–4). The rankings for the top three most prevalent bacterial pathogens were different across age groups with the rank being (1) *S pneumoniae*, (2) *M pneumoniae*, and (3) *H influenzae* for children; (1) *M pneumoniae*, (2) *S pneumoniae*, and (3) *H influenzae* for adolescents; *(1) K pneumoniae, (2) P aeruginosa, (3) S pneumoniae* for adults*;* and (1) *P aeruginosa*, (2) *K pneumoniae*, and (3) *S pneumoniae* for older adults (appendix 2 pp 3–4). Generally, there was more positive bacterial detection than viral detection in adults (23·64% [342/1447] vs 20·33% [529/2602]) and older adults (22·96% [451/1964] vs 19·12% [525/2746]); in contrast, children had more viral than bacterial positive detection (50·82% [3390/6671] vs 34·37% [544/1583]), and similar rates were seen for viral and bacterial positive detection in adolescents.

**Figure 1:**
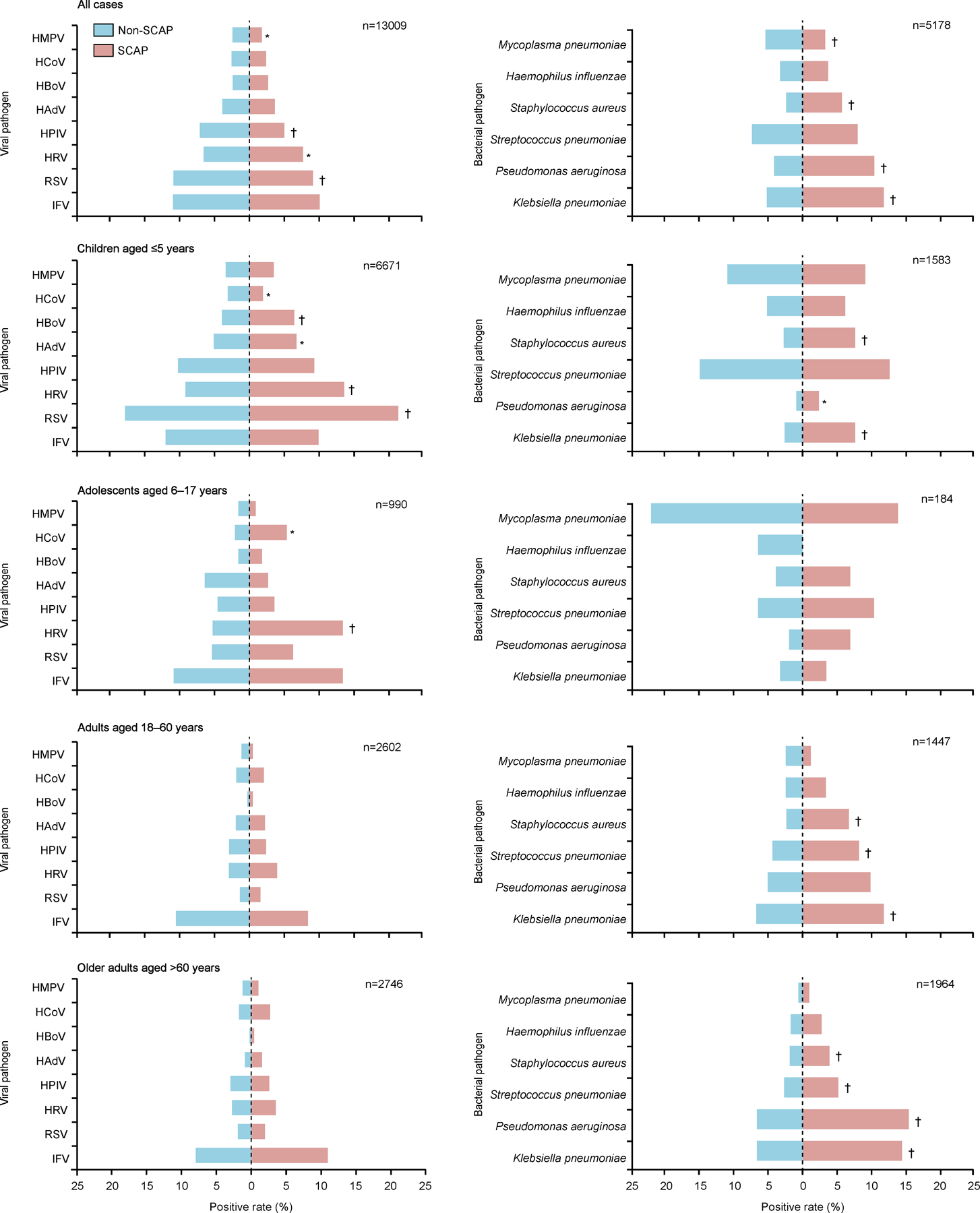
Comparison of the positive rates of eight viral and six bacterial pathogens in patients with SCAP and non-SCAP in mainland China, 2009‒20 The positive rate of each pathogen among 13 009 patients with pneumonia (2695 SCAP and 10 314 non-SCAP) tested for all the eight viral pathogens and among 5178 patients with pneumonia (1405 SCAP and 3773 non-SCAP) tested for all the six bacterial pathogens was compared for different age groups. The length of the red bar indicates the positive rate of SCAP and the length of the blue bar indicates the positive rate of non-SCAP. Positive rate was calculated by taking the positive number of each pathogen as the numerator and the number of CAP tested as denominator. The significant difference of the positive rate (χ^2^ test or Fisher’s exact test) is indicated. HAdV=human adenovirus. HBoV=human bocavirus. HCoV=seasonal human coronavirus. HMPV=human metapneumovirus. HPIV=human parainfluenza virus. HRV=human rhinovirus. IFV=influenza virus. RSV=respiratory syncytial virus. SCAP=severe community acquired pneumonia. *p<0·05. †p<0·01.

2695 patients with SCAP were tested for all the eight viral pathogens : 34·58% (932) had at least one positive detection, with the highest rate determined in children (57·19% [561/981]), followed by 35·71% (40/112) in adolescents, 19·59% (124/633) in adults, and 21·36% (207/969) in older adults (appendix 2 pp 5–6). Significantly higher positivity rates were seen in SCAP than in non-SCAP for children (57·19% [561/981] vs 49·72% [2829/5690]; p<0·0001) and older adults (21·36% [207/969] vs 17·90% [318/1777], p=0·027), but not for other age groups. In SCAP, IFV had the highest positive rate (10·06% [271/2695]), followed by RSV (9·09% [245/2695]), HRV (7·68% [207/2695]), HPIV (5·01% [135/2695]), HAdV (3·64% [98/2695]), HBoV (2·67% [72/2695]), HCoV (2·37% [64/2695]), and HMPV (1·78% [48/2695]); the same order was also seen for CAP. The rankings for the top three most prevalent viral pathogens were different across age groups with the rank being (1) RSV, (2) HRV, and (3) IFV for children; (1) IFV, (2) HRV, and (3) RSV for adolescents; (1) IFV, (2) HRV, and (3) HPIV for adults; and (1) IFV, (2) HRV, and (3) HCoV for older adults (figure 1).

1405 patients with SCAP were tested for all the six bacterial pathogens : 35·59% (500) had at least one positive detection, with the highest rate determined in children (40·47% [138/341]), followed by 37·93% (11/29) in adolescents, 34·30% (212/618) in older adults, and 33·33% (139/417) in adults (appendix 2 pp 5–6). Significantly higher bacterial positive rates were seen in the SCAP than the non-SCAP group (35·59% [500/1405] vs 23·91% [902/3773]; p<0·0001). *K pneumoniae* had the highest positive rate in SCAP (11·74% [165/1405]), followed by *P aeruginosa* (10·39% [146/ 1405]), *S pneumoniae* (7·97% [112/1405]), *S aureus* (5·69% [80/1405]), *H influenzae* (3·70% [52/1405]), and *M pneumoniae* (3·27% [46/1405]), differing from the ranking in CAP as a whole, with the SCAP versus non-SCAP difference being significant for *K pneumoniae* (11·74% [165/1405] vs 5·17% [195/3773]), *P aeruginosa* (10·39% [146/1405] vs 4·11% [155/3773]), and *S aureus* (5·69% [80/1405] vs 2·36% [89/3773], all p<0·0001), which was also seen in children and old adults. The rankings for the top three most prevalent bacterial pathogens were different across age groups with the rank being (1) *S pneumoniae*, (2) *M pneumoniae*, and (3) *K pneumoniae* for children; (1) *M pneumoniae*, (2) *S pneumoniae*, and (3) *S aureus* for adolescents; (1) *K pneumoniae*, (2) *P aeruginosa*, and (3) *S pneumoniae* for adults; and (1) *P aeruginosa*, (2) *K pneumoniae*, and (3) *S pneumoniae* for older adults (figure 1). Clinical features among patients with CAP infected with different respiratory pathogens are in appendix 2 (pp 7–8).

3552 patients with CAP were tested for all 14 viral and bacterial pathogens. Of these, co-infections were determined in 798 (22·47%) patients, with 11·40% (405/3552) being viral–bacterial co-infection, 7·43% (264/3552) with viral–viral co-infections, and 3·63% (129/3552) with bacterial–bacterial co-infections. The bacterial–bacterial co-infection rate increased with age and viral–viral co-infection and viral–bacterial co-infection decreased with age (appendix 2 p 9). RSV–HRV co-infection had the highest positive rate (2·67% [95/3552]), followed by RSV–S pneumoniae (1·60% [57/3552]), RSV–IFV (1·58% [56/3552]), HRV–S pneumoniae (1·49% [53/3552]), and RSV–HPIV (1·46% [52/3552]; appendix 2 p 15). The viral–viral and viral–bacterial co-infection rates were higher in the cold than the warm season (appendix 2 p 9). The rankings for the top three most prevalent co-infections in the cold season were (1) RSV–HRV, (2) RSV–IFV, and (3) RSV–S pneumoniae, in contrast to (1) RSV–HRV, (2) HRV–HPIV, and (3) HRV–S pneumoniae in the warm season (appendix 2 p 16)

**Figure 2:**
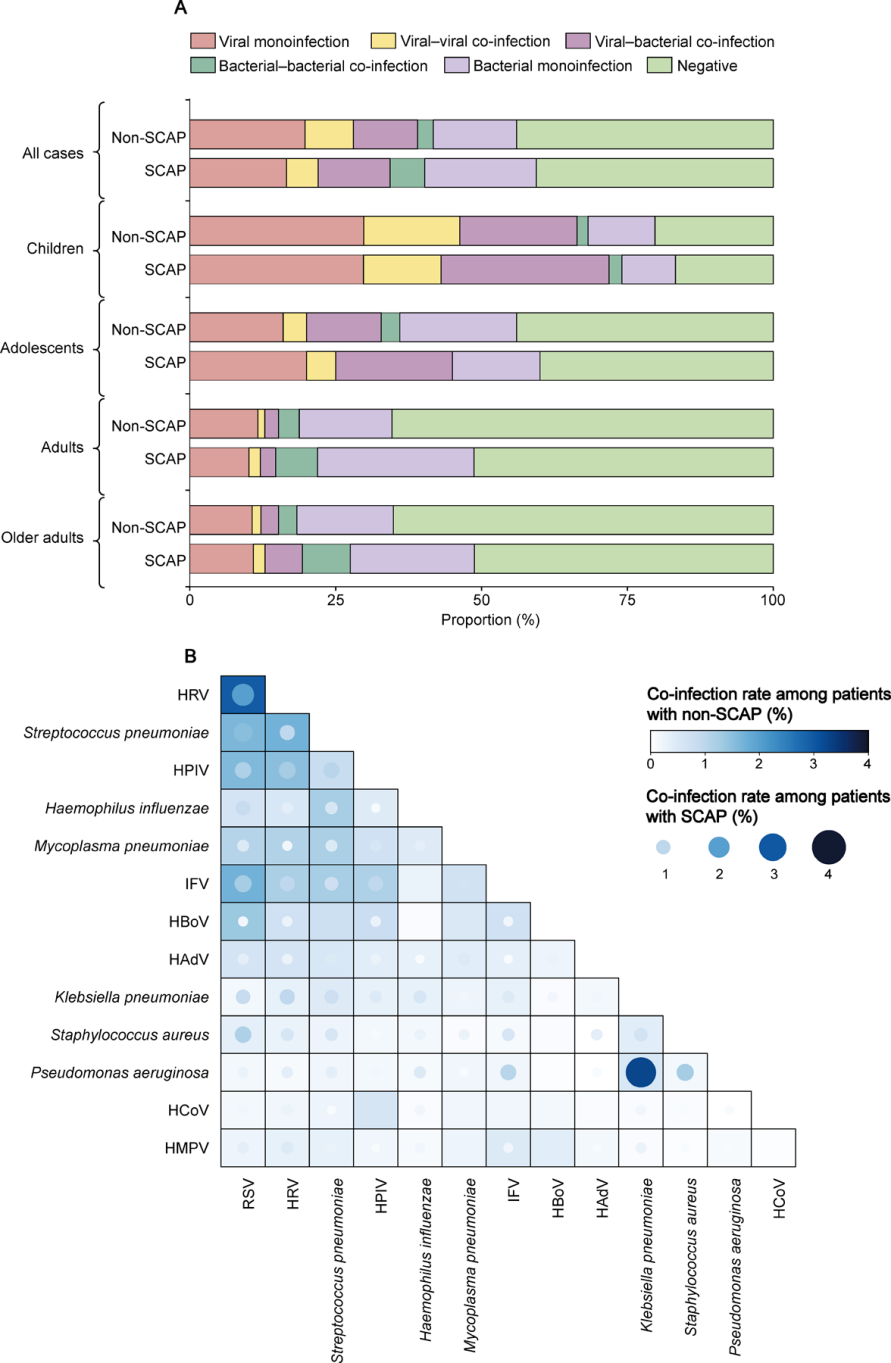
Prevalence of pathogens in mono infection and co-infection in patients with SCAP and non-SCAP in mainland China, 2009‒20 (A) Positive proportion of viruses, viral-viral co-infections, viral-bacteria co-infections, bacterial-bacterial co-infections, and bacteria in different age groups. (B) Heatmap of the co-infection rate of respiratory pathogens. The grid colour represents the co-infection rate of respiratory pathogens among patients with non-SCAP, and the dot colour represents the co-infection rate of respiratory pathogens among patients with SCAP. Bigger size and darker colour of the circles indicates higher co-infection rates between the pair of pathogens. HAdV=human adenovirus. HBoV=human bocavirus. HCoV=seasonal human coronavirus. HMPV=human metapneumovirus. HPIV=human parainfluenza virus. HRV=human rhinovirus. IFV=influenza virus. SCAP=severe community acquired pneumonia.

1046 patients with SCAP were tested for all 14 pathogens and 248 (23·71%) had co-infections, with 12·33% (129/1046) being viral–bacterial co-infection, 5·93% (62/1046) being bacterial–bacterial co-infection, and 5·45% (57/1046) being viral–viral co-infection. A higher co-infection rate was observed in SCAP than in non-SCAP for adults (11·76% [36/306] vs 7·09% [42/592]) and older adults (16·58% [67/404] vs 7·67% [51/665]). A higher bacterial–bacterial co-infection rate was observed in SCAP than in non-SCAP among adults (7·19% [22/306] vs 3·55% [21/592]) and older adults (8·17% [33/404] vs 3·16% [21/665]). A higher viral–bacterial co-infection rate was observed in SCAP than in non-SCAP among children (28·80% [91/316] vs 20·11% [226/1124]), and older adults (6·44% [26/404] vs 3·01% [20/665]). No difference of viral–viral co-infection rate was observed between SCAP and non-SCAP for any age group (figure 2A; appendix 2 p 10).

The most common bacterial co-infections were *K pneumoniae-P aeruginosa*, *S aureus-P aeruginosa*, and *K pneumoniae-S pneumoniae*; all the rates were significantly higher among patients with SCAP than patients with non-SCAP. Viral-bacterial co-infection primarily occurred among *S pneumoniae*, RSV, and HRV, with co-infection rate of RSV-*S aureus*, IFV*-P aeruginosa*, and HRV*-K pneumoniae*, significantly higher among patients with SCAP than non-SCAP. Viral-viral co-infection primarily occurred for RSV-HRV, HPIV-HRV, and RSV-IFV; all were higher in patients with non-SCAP than with SCAP (figure 2B). The top ranking co-infection was different across age groups RSV-HRV in children, *S pneumoniae*-*H influenzae* in adolescents, *S pneumoniae*-*H influenzae* in adults, and *K pneumoniae*-*P aeruginosa* in older adults (appendix 2 p 9).

**Figure 3:**
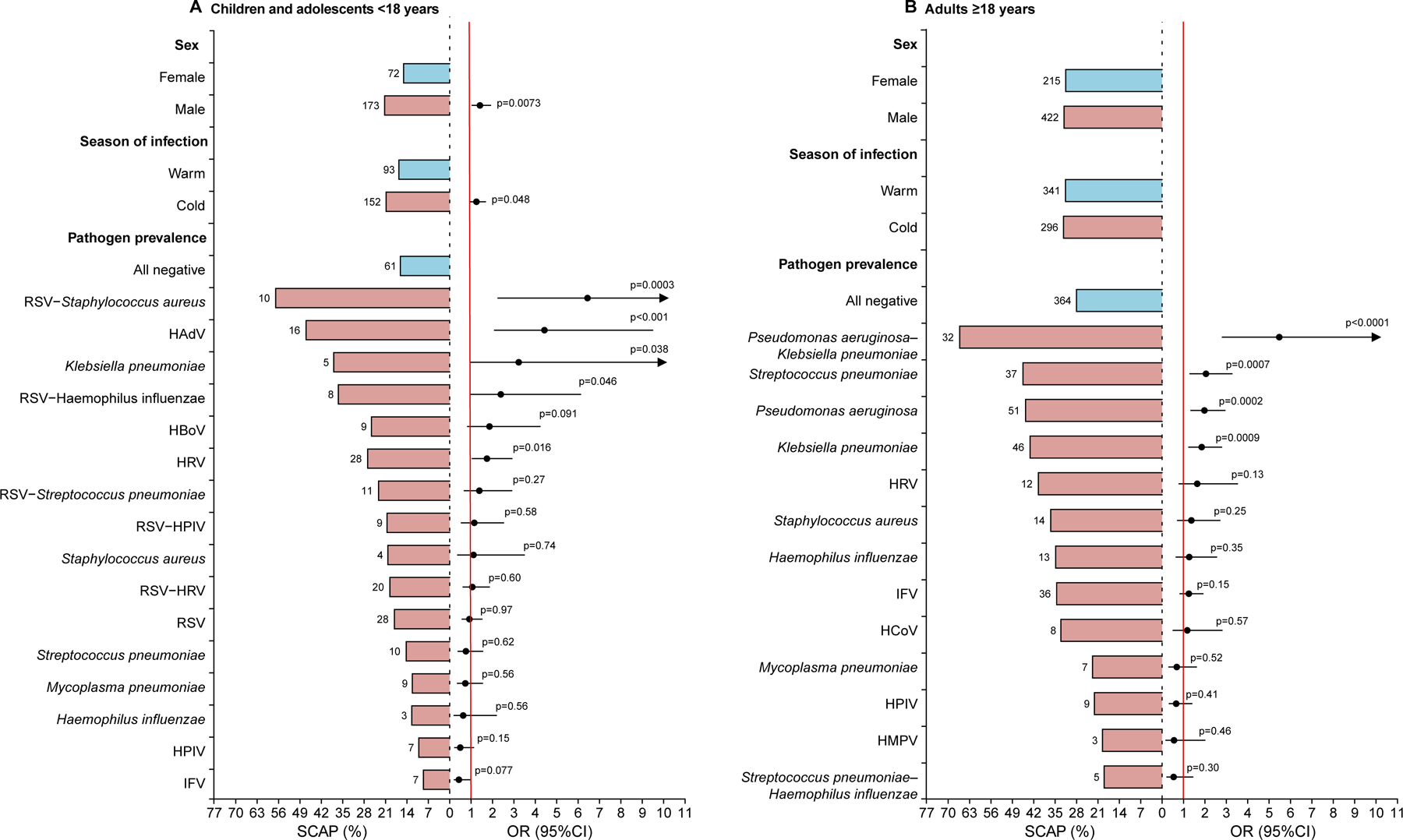
Adjusted OR for SCAP The numbers next to the bars represent the number of patients with SCAP in each group. The lengths of the bars indicate the incidence rate of SCAP, with blue bar representing the reference groups. The black points are the adjusted ORs for SCAP and the black error bars are the 95% CIs, and the arrow indicates that the 95% CI is out of the graph. (A) Incidence and adjusted OR for SCAP in children and adolescents younger than 18 years. (B) Incidence and adjusted OR for SCAP in adults aged 18 years or older. HAdV=human adenovirus. HBoV=human bocavirus. HCoV=seasonal human coronavirus. HMPV=human metapneumovirus. HPIV=human parainfluenza virus. HRV=human rhinovirus. IFV=influenza virus. RSV=respiratory syncytial virus. SCAP=severe community acquired pneumonia.

The association with SCAP development, ICU admission and death were explored for patients younger than 18 years. An increased incidence of SCAP was associated with being male (OR 1·55, 95% CI 1·12–2·12); having disease onset in the cold season (1·37, 1·00–1·86); and a positive detection for RSV-*S aureus* (7·07, 2·45–20·40), HAdV (4·86, 2·27–10·44), *K pneumoniae* (3·54, 1·07–11·69), RSV-*H influenzae* (2·62, 1·02–6·74), and HRV (1·91, 1·13–3·22) by multivariate logistic regression analysis (figure 3A; appendix 2 p 12). For adults aged 18 years or older, a significant association was observed for positive detection of *P aeruginosa*-*K pneumoniae* (6·01, 3·06–11·80), *S pneumoniae* (2·25, 1·40–3·61), *P aeruginosa* (2·17, 1·45–3·25), and *K pneumoniae* (2·03, 1·34–3·08) (figure 3B; appendix 2 p 12). Positive detection of *P aeruginosa*-*K pneumoniae, S aureus*, *K pneumoniae*, *P aeruginosa*, or *S pneumoniae* was significantly associated with increased risk of ICU admission (appendix 2 p 13). Increased incidence of death was significantly associated with positive detection of *P aeruginosa*-*K pneumoniae*, *S pneumoniae*, and IFV (appendix 2 p 14).

**Figure 4:**
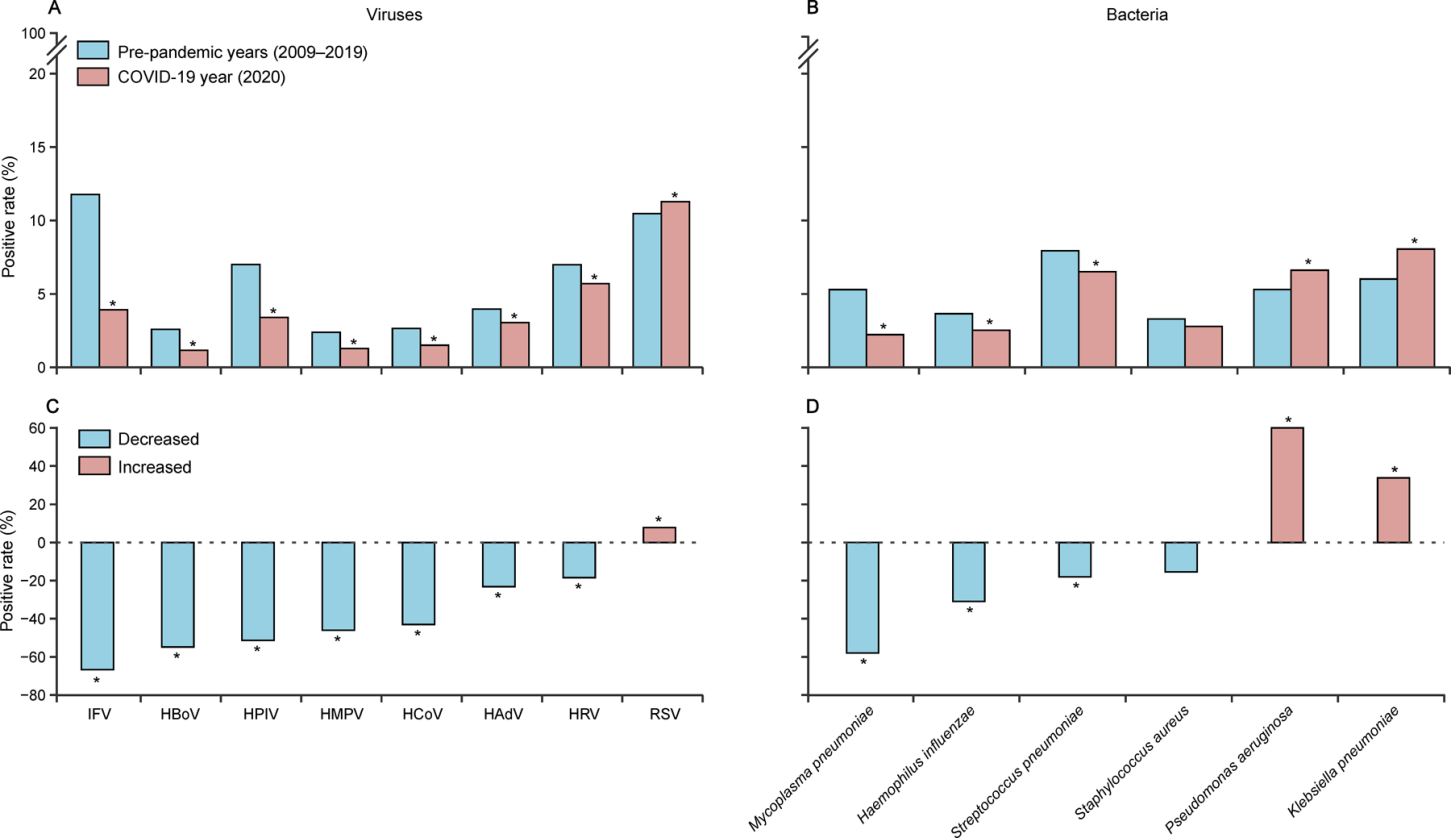
Comparison of age-standardised test positive rates of pathogens and their percent changes between the pre-pandemic years (2009‒19) and the first COVID-19 pandemic year (2020) (A) Age-standardised positive rates of eight viruses detected in patients with CAP during pre-pandemic years and the first COVID-19 pandemic year in mainland China. (B) Percent change of test positive rates of eight viruses detected. (C) Age-standardised positive rates of six bacteria detected in patients with CAP during pre-pandemic years and the first COVID-19 pandemic year. (D) Percent change of test positive rate of six bacteria detected. Statistical significance was based on χ^2^ test or Fisher’s exact test. HAdV=human adenovirus. HBoV=human bocavirus. HCoV=seasonal human coronavirus. HMPV=human metapneumovirus. HPIV=human parainfluenza virus. HRV=human rhinovirus. IFV=influenza virus. RSV=respiratory syncytial virus. CAP=community acquired pneumonia. *p<0·01.

Positive detection was compared between the pre-pandemic (2009–19) and the pandemic year (2020). All tested viral pathogens except for RSV had annual cumulative positive rate significantly decreased. The largest decrease in the positive rate was observed for IFV (–66·67%), followed by HBoV (–54·83%), HPIV (–51·36%), HMPV (–46·03%), HCoV (–43·02%), HAdV (–23·17%), and HRV (–18·43%). A significantly increased positive rate was observed for RSV (from 10·46% to 11·28%, p <0·0001; figure 4A, C). The positive rate of three of the six tested bacterial pathogens decreased significantly. The largest decrease of positive rate was observed for *M pneumoniae* (–57·92%), followed by *H influenzae* (–30·87%), and *S pneumoniae* (–17·99%). However, significantly increased positive rates were observed for *P aeruginosa* and *K pneumoniae* (figure 4B, D). The overall co-infection rate also decreased significantly from 24·28% to 11·29%. The largest decrease was observed for RSV-HRV, followed by *S pneumoniae*-*H influenzae*, HRV-*M pneumoniae*, HRV-HPIV, and IFV-*M pneumoniae*. However, co-infection rates of *K pneumoniae*-*S aureus*, HAdV-HMPV, and *K pneumoniae*-*P aeruginosa* increased above historical levels (appendix 2 p 17). Comparison of incidence of SCAP in different age groups in each season is in appendix 2 (p 18).

## Discussion

In this comprehensive national surveillance of CAP spanning 12 years in China, we describe the causal and epidemiological characteristics of CAP, identify patterns of infection and co-infection of 14 viral and bacterial pathogens among patients with SCAP and non-SCAP and explore differences by age and season. We further examine the association between clinical outcomes (SCAP and ICU admission) and these infection and co-infection patterns, as well as documenting the effects introduced by COVID-19 pandemic control policies.

We found that RSV dominated in all viral CAP and SCAP subgroups for children; and the leading bacterial pathogens in the CAP and SCAP subgroups was *S pneumoniae* for children*, M pneumoniae* for adolescents*, K pneumoniae* for adults, and *P aeruginosa* for older adults. We found that bacterial infections (23·64%) were more frequently found than viral infections (20·33%) in adults with CAP, viral detection (50·82% for viruses vs 34·37% for bacteria) was more frequently found in children, and viral and bacterial positive rates were comparable for adolescents. Our data also showed that positive detection rates of both viral and bacterial pathogens was higher in children with CAP, and both had shown a decreasing trend with the increase of age (appendix 2 pp 3‒4). It has been suggested that age-dependent factors might help explain these epidemiological patterns, such as frequent close contact among children in nurseries or schools leading torespiratory pathogen transmission..^14^ These age-dependent patterns could also be related to increased susceptibility to infections during childhood, due to lower quantitative and functional specific immune responses generated and less pre-existing immunological memory in children than in adults.^15^ Children show lower levels, reduced affinity, and diversity of T-cell dependent antibody responses compared with adults, which might also be responsible for these age-dependent patterns.^16^

Most previous studies did not have enough patients to determine the co-infection pattern within SCAP by age. Here we showed that for both children and older adults higher rates of viral-bacterial co-infection were found in SCAP over non-SCAP; however, for adults, only higher bacterial-bacterial co-infection was observed. This age difference might reflect a more important pathogenic role of viral infection among the oldest and younger age groups. Viral-viral co-infection was not over-represented in SCAP for any of the age groups. This finding is consistent with the phenomenon called viral interference, whereby one virus blocks the growth of another virus in the context of multiple viral mixed infection.^17^ Although it has been intensively studied, the mechanism underlying viral interference differs among viruses. It is generally suggested that direct interactions of viral genes or gene products, indirect interactions from alterations in the host environment, and immunological interactions might be variously responsible.^18^

We found more viral-bacterial co-infections than viral-viral and bacterial-bacterial co-infections, with even higher viral-bacterial co-infection rate in SCAP than non-SCAP, for which various complex interactions might be hypothesised. Virus infection might induce airway damage, further promoting bacterial adherence, and cause dysregulation of host immune system, both processes promote bacterial growth.^19,20^ Conversely, bacterial infection might predispose to viral infections by altering the ease at which viruses propagate and infect within the respiratory system.^21^ These bi-directional effects underlie the pathogenesis of viral-bacterial co-infection, contributing to poorer clinical outcomes including prolonged hospital stay and higher mortality rates.^21^

We also showed an age difference in co-infection patterns, and specifically in the SCAP group. Higher co-infection rate of RSV-HRV was seen in children, compared with a higher co-infection rate of *K pneumoniae*-*P aeruginosa* in adults and older adults. Co-infection of RSV-*H influenzae* and RSV-*S aureus* in children and adolescents, and *P aeruginosa-K pneumoniae* in adults aged 18 years and older was associated with SCAP. The high susceptibility to SCAP for males was determined in children and adolescents, which was consistent with previous findings,^22^ which is probably due to immune response, airway mechanics, and narrow airway in this population.^23^ This finding is also consistent with findings among hospitalised school-age children in Greece, in which the viral-bacterial co-infection rate was higher than that of bacterial-bacterial co-infection or viral-viral co-infection in pneumonia.^24^ Viral-bacterial co-infection pathophysiology is multifactorial and related to changes in the anatomy of the lung allowing for bacterial invasion and production of virulence factors that facilitate co-infection or secondary infection.^19,21^ Moreover, bacterial co-infection has been well described with influenza in conjunction with *S aureus*, *S pneumoniae*, *H influenza, and Streptococcus hemolyticus*.^25^ Other respiratory viruses are well described as underlying causes of pneumonia including HRV, RSV, HMPV, and HPIV.^21^

Climatic factors have been widely suggested to influence the interaction among host, pathogen, and environment, contributing to the seasonality of respiratory pathogens. In general, positive detection of viral pathogens was higher in the cold season than warm season, which might be associated with the increased crowding indoor activity, vitamin D deficiency due to lower sun exposure during the cold seasons, and lower temperatures increasing the stability of virions outside the body.^26^ For instance, RSV and IFV had similar seasonal patterns, all favouring circulation in lower temperature and vapour pressure.^27,28^ Bacterial pathogens largely showed no significant seasonal variation. These different affinities to climate were determinant of the complex infection and co-infection pattern as a response to seasonal change, which had been rarely studied.

The study was subject to the major limitations that causal relationships cannot be determined from positive detection of the pathogens, and it is difficult to judge whether co-infection was a cause or consequence of CAP. Limitations, inherent to causal surveillance (ie, multiple sample types and multiple tests with varying sensitivity and specificity) will also have affected this work. The reliance on PCR testing also presents challenges because it could represent coincidental infection or a pathogenic role, thus distinguishing prolonged shedding from colonisation was difficult. Measurement of background prevalence of asymptomatic infection in a control group might help to clarify the causal associations in future work. Finally, baseline characteristics, such as comorbidity, BMI, and drug combination are also potential factors that can affect the infection pattern of respiratory pathogens and the severity of CAP disease. Measuring all these factors might be used in combination with the pathogen co-infection patterns spectrum identified here to further identify risk factors associated with SCAP in future work.

It is hoped that the patterns seen in the current study, although descriptive, are of wide interest and might help better identify target pathogens for applying diagnostic assays for more accurate diagnoses, treatment, and control. These findings might also enrich the evidence base for improving guidelines for the prescription of antibiotics and age and pathogen targeted vaccination as well as promoting schemes for better health-care use to collectively help reduce SCAP in populations at high risk in China and beyond.

## Supplementary Material

Supplementary Appendix

## Figures and Tables

**Table:** Demographic characteristics of patients with CAP in mainland China, 2009‒20

	Total (N=18 807)	Non-SCAP (n=15 036)	SCAP (n=3771)	p value*
Sex	∙∙	∙∙	∙∙	<0·0001
Female	6863 (36·49%)	5670 (37·71%)	1193 (31·64%)	∙∙
Male	11 944 (63·51%)	9366 (62·29%)	2578 (68·36%)	∙∙
Age group	∙∙	∙∙	∙∙	<0·0001
Children, ≤5 years	8240 (43·81%)	7064 (46·98%)	1176 (31·19%)	∙∙
Adolescents, 6**‒**17 years	1301 (6·92%)	1136 (7·56%)	165 (4·38%)	∙∙
Adults, 18**‒**60 years	4942 (26·28%)	3846 (25·58%)	1096 (29·06%)	∙∙
Older adults, >60 years	4324 (22·99%)	2990 (19·89%)	1334 (35·38%)	∙∙
Season of infection†	∙∙	∙∙	∙∙	0·1233
Cold	10 824 (57·55%)	8696 (57·83%)	2128 (56·43%)	
Warm	7983 (42·45%)	6340 (42·17%)	1643 (43·57%)	
Case type	∙∙	∙∙	∙∙	<0·0001
Inpatient	14 479 (76·99%)	11 168 (74·28%)	3311 (87·80%)	∙∙
Outpatient	4328 (23·01%)	3868 (25·72%)	460 (12·20%)	∙∙
Intensive care unit admission	∙∙	∙∙	∙∙	<0·0001
Yes	2183 (11·61%)	917 (6·10%)	1266 (33·57%)	∙∙
No	15 614 (83·02%)	13 157 (87·5%)	2457 (65·16%)	∙∙
Unknown	1010 (5·37%)	962 (6·40%)	48 (1·27%)	∙∙
Outcome				<0·0001
Died	150 (0·80%)	56 (0·37%)	94 (2·49%)	∙∙
Alive	15 705 (83·51%)	12 629 (83·99%)	3076 (81·57%)	∙∙
Unknown	2952 (15·70%)	2351 (15·64%)	601 (15·94%)	∙∙

Date are n (%). CAP=community-acquired pneumonia. SCAP=severe community-acquired pneumonia.

* p value was compared between non-SCAP group and SCAP group.

† For each patient with CAP, the onset date was divided into cold season or warm season based on the climate characteristics of each sentinel city, that is, the 6 months with the highest monthly average temperature was the hot season, and other months were the cold season.

## Data Availability

Relevant data that support the findings of this study and model results generated as part of this study are publicly available within the paper and in appendix 2. Raw data are not publicly available due to restrictions by the data provider, which were used under license for the current study, but are available upon reasonable request to both corresponding authors and with permission from the data providers (L-PW and WL).

## Research in context

### Evidence before this study

We searched PubMed for articles published from Jan 1, 2009, to July 20, 2022, using search terms “community-acquired pneumonia” or “pneumonia” AND “etiology/pathogen/viruses/virus/viral/bateria/bacterium/bacterial” AND “China”. No language restrictions were applied to this search. Among the 860 articles retrieved from the preliminary screening, 60 articles reported detection of community-acquired pneumonia in China during 2009‒21. Of these, 30 articles reported single pathogen detection that involved any of influenza virus, human rhinovirus (HRV), human bocavirus, *Mycoplasma pneumoniae*, *Klebsiella pneumoniae*, and *Streptococcus pneumoniae*. A further 30 articles reported detection of multiple pathogens, including nine articles focusing on viral infections, three articles focusing on bacterial infections, and 18 articles investigating both viral and bacterial pathogens. None of co-infection patterns in community acquired pneumonia (CAP) had been investigated at the national level. The infection and co-infection patterns were largely attributed to the different affinity of respiratory pathogens at the individual level or related to climatic conditions at the population level. Most studies did not have enough patients to determine patterns by age. How infection and co-infection might be affected by seasonal change was also poorly understood.

### Added value of this study

This study provided unprecedented causal and epidemiological detail of 18 807 all-age patients with CAP drawn from a hospital network covering 30 provinces of China from January, 2009, to December, 2020. A total of 14 respiratory pathogens and their infection or co-infection patterns were diagnosed, giving an unparalleled insight into the age-specific cause of CAP. We determined a more frequent bacterial infection than viral infection in adults (aged 18‒60 years) and older adults (aged >60 years), and more frequent viral infection in children (aged ≤5 years). We observed higher rates of viral positive detection, bacterial positive rate, as well as viral-bacterial co-infection in severe CAP (SCAP) than in non-SCAP, but only among children and older adults. The top-ranking positive detection of viral pathogens, bacterial pathogens, and their co-infection were presented among all patients with CAP and SCAP separately, in an age, sex, and season-dependent manner. By performing a multivariate analysis, positive detection with human adenovirus, HRV, *K pneumoniae*, co-infections of respiratory syncytial virus (RSV)–*Haemophilus influenzae* or RSV–*Staphylococcus aureus* were found to be significantly associated with increased incidence of SCAP in children and adolescents (aged <18 years), while positive detection of *Pseudomonas aeruginosa*, *K pneumoniae*, *S pneumoniae,* or *P aeruginosa*-*K pneumoniae* co-infection were associated with increased incidence of SCAP in adults aged 18 years or older.

### Implications of all the available evidence

Our findings have many important implications for guiding medical practice and care for patients with pneumonia across various age groups, particularly in applying correct diagnostic assays to help identify those most at risk of SCAP development. Our findings also help for a better understanding of the true demand and probable age-spectrum for delivery, of putative pneumoniae vaccines in China.
