# Supplementary material for: Infection and co-infection patterns of community-acquired pneumonia in patients of different ages in China from 2009‒2020: a national surveillance study
Source: Lancet Microbe. Author manuscript; Available in PMC 2025 Oct 11. (PMC12514336; doi:10.1016/S2666-5247(23)00031-9)
Supplement: Supplementary Appendix [file NIHMS2109448-supplement-Supplementary_Appendix.docx]

**Supplementary Appendix:**

**Supplement to: Infection and coinfection patterns of community acquired pneumonia in patients of different ages in China from2009‒2020: a national surveillance study**

**Table of Contents**

| **Page** | **Items** |
| --- | --- |
| 1–2 | Supplementary Methods |
| 3–4 | Supplementary table 1. Positive rate of respiratory pathogens among patients with CAP by age, gender, case type, ICU admission, and clinical outcome, and season of infection in Chinese mainland, 2009‒2020. |
| 5–6 | Supplementary table 2. Positive rate of respiratory pathogens among patients with SCAP and non-SCAP by age group, gender, case type, and season of infection in Chinese mainland, 2009‒2020. |
| 7–8 | Supplementary table 3. Clinical features among CAP patients infected with different respiratory pathogens. |
| 9 | Supplementary table 3. Co-infection rate of CAP patients by age. |
| 10 | Supplementary table 4. Co-infection rate compared between SCAP and non-SCAP patients by age. |
| 11 | Supplementary table 5. Coinfection patterns for different age groups between SCAP and non-SCAP patients. |
| 12 | Supplementary table 6. The association between SCAP and age, sex, season of infection, pathogen prevalence. |
| 13 | Supplementary table 7. The association between ICU admission of patients and age, sex, season of infection, pathogen prevalence for adults≥18 years. |
| 14 | Supplementary table 8. The association between death of patients and pathogen prevalence by conditional logistic regression in adults ≥18 years. |
| 15 | Supplementary figure 1. Prevalence of pathogens in mono infection and coinfection determined in patients with CAP in the Chinese mainland, 2009‒2020. |
| 16 | Supplementary figure 2. Comparison of coinfections rate between cold and warm season. |
| 17 | Supplementary figure 3. Comparison of coinfections rate between pre-pandemic years (2009‒2019) and COVID-19 pandemic year (2020). |
| 18 | Supplementary figure 4. Comparison of the incidence of SCAP in different age groups in each season between pre-pandemic years (2009‒2019) and COVID-19 pandemic year (2020). |
| 19–20 | Supplementary list of authors and their affiliations |

**Supplementary Methods**

*Case definition of acute respiratory infection*

Acute respiratory infection was defined as presenting fever or chills, leukocytosis or leukopenia, acute respiratory illness, defined as newly developed cough or sputum production, chest pain, dyspnea, tachypnea, and abnormal lung examination)

*Case definitions of severe community acquired pneumonia (CAP)*

SCAP in patients of <18 years was defined as pneumonia plus hypoxemia (maintained SaO_2_ <92% in air) or increasing respiratory and pulse rates with clinical evidence of respiratory distress and exhaustion with or without raised PaCO_2_, according to the guidelines for the management of common childhood illnesses from WHO^1,2^. Based on the Infectious Diseases Society of America (IDSA)/American Thoracic Society (ATS) criteria^3,4^, SCAP in patients of ≥18 years was defined as CAP patients with either one major criterion or three or more minor criteria: (1) Major criteria include either septic shock with need for vasopressors or respiratory failure requiring mechanical ventilation; (2) Minor criteria include respiratory rate ≥30 breaths/min, PaO_2_/FiO_2_ ratio ≤250, multilobar infiltrates, confusion/disorientation, uremia (blood urea nitrogen ≥20 mg/dL), leukopenia (white blood cell [WBC] count <4 000 cells/µl), thrombocytopenia (platelet count [PLT] <100 000 /µl), hypothermia (core temperature <36℃), or hypotension requiring aggressive fluid resuscitation.

*Supervision of the study*

In addition, all sentinel hospitals used a standard operating protocol (SOP) of surveillance that included guidelines for patient enrollment, specimen collection, laboratory testing, data recording, and management, developed by China CDC, which had been published^5^. All the sentinel hospitals had undergone training to be qualified for recruiting patients, sample collection, and test following the SOP, before the surveillance was started. Pre-study training, whole-procedure supervision, monthly enrollment reports, data audits, and annual study-site visits were conducted to ensure uniform procedures were followed as guided among the study sites and unchanged across the surveillance years.

*Statistical analysis*

Descriptive statistics included frequencies (proportions) for categorical variables, and medians with interquartile ranges (IQR) for continuous variables. Chi-square test or Fisher’s extract test was applied for the inter-group comparison. A binary logistic regression model was applied to examine variables that were related to SCAP and ICU admission, with sex, age, season of infection, and infection of pathogens included as explanatory variables. Infection or coinfection of pathogens with total positive number ≤10 was not included in the model. Interaction between age (juveniles <18 years, adults ≥18 years) and other candidate variables was introduced in the model given their different host response to respiratory pathogens. Multivariate analysis was performed by including all variables with a p-value <0·10 from the univariate analysis as covariates. We estimated OR and the 95% CI using maximum likelihood methods. The ICU admission as the preferred treatment for patients with more severe patients, was also used as an outcome variable for adults ≥18 years, while juveniles <18 years was not included in this analysis due to small sample of ICU admission (306 cases). In addition, a conditional logistic regression was performed to examine the potential association between death outcome and the positive tests for respiratory pathogens for adults ≥18 years (3 death cases for juveniles <18 years were excluded), by including all 51 lethal cases and 5 times controls randomly selected from all recovered cases after matching their age, sex, and season of infection. All the statistical analysis was performed using R version 4·1·1. P-value of <0·05 was considered statistically significant.

**References**

1. World Health Organization. Guidelines for the Management of Common Childhood Illnesses (2nd edition). Available at: https://www.ncbi.nlm.nih.gov/books/NBK154448/#ch4.s8.
2. Wang ZB, Ren L, Lu QB, et al. The impact of weather and air pollution on viral infection and disease outcome among pediatric pneumonia patients in Chongqing, China, from 2009 to 2018: a prospective observational study. *Clin Infect Dis* 2021; **73**(2): e513-e22.

3. Mandell LA, Wunderink RG, Anzueto A, et al. Infectious Diseases Society of America/American Thoracic Society consensus guidelines on the management of community-acquired pneumonia in adults. *Clin Infect Dis* 2007; **44 Suppl 2**(Suppl 2): S27-72.

4. Wongsurakiat P, Chitwarakorn N. Severe community-acquired pneumonia in general medical wards: outcomes and impact of initial antibiotic selection. *BMC Pulm Med* 2019; **19**(1): 179.

5 Li MF., Ren, LL., Yu HJ. Pathogen surveillance and detection techniques: febrile respiratory syndrome (in Chinese). Sun Yat-Sen University Press: Guangzhou, 2017.

**Supplementary table 1. Positive rate of respiratory pathogens among patients with CAP by age, gender, case type, ICU admission, clinical outcome, and season of infection in Chinese mainland, 2009‒2020.**

|  | **Total** |  | **Age group** | | | | |  | **Sex** | | |
| --- | --- | --- | --- | --- | --- | --- | --- | --- | --- | --- | --- |
|  |  |  | **Children**  **(≤ 5 years)** | **Adolescents**  **(6‒17 years)** | **Adults**  **(18‒60 years)** | **The elderly**  **(>60 years)** | **P value** |  | **Male** | **Female** | **P value** |
| Virus | 4 766/13 009  (36·64) |  | 3 390/6 671  (50·82) | 322/990  (32·53) | 529/2 602  (20·33) | 525/2 602  (19·12) | <0·0001 |  | 2 984/8 033  (37·15) | 1 782/4 976  (35·81) | 0·1293 |
| IFV | 1 397 (10·74) |  | 780 (11·69) | 110 (11·11) | 260 (9·99) | 247 (8·99) | 0·0008 |  | 880 (10·95) | 517 (10·39) | 0·3259 |
| *IFV-A* | 767 (39·93) |  | 391 (28·83) | 43 (36·75) | 160 (72·40) | 173 (76·21) | <0·0001 |  | 481 (39·65) | 286 (40·40) | 0·7857 |
| *IFV-B* | 361 (18·79) |  | 247 (18·22) | 35 (29·91) | 38 (17·19) | 41 (18·06) | 0·0167 |  | 215 (17·72) | 146 (20·62) | 0·1317 |
| *IFV-C* | 14 (1·33) |  | 6 (1·16) | 1 (1·16) | 4 (1·81) | 3 (1·32) | 0·8886 |  | 10 (1·56) | 4 (0·97) | 0·5909 |
| *IFV-untyped* | 99 (9·42) |  | 54 (10·44) | 10 (11·63) | 21 (9·50) | 14 (6·17) | 0·2676 |  | 66 (10·31) | 33 (8·03) | 0·2591 |
| RSV | 1 366 (10·50) |  | 1 222 (18·32) | 54 (5·45) | 37 (1·42) | 53 (1·93) | <0·0001 |  | 873 (10·87) | 493 (9·91) | 0·0879 |
| *RSV-A* | 625 (40·90) |  | 563 (40·10) | 20 (43·48) | 19 (55·88) | 23 (52·27) | 0·1111 |  | 400 (40·20) | 225 (42·21) | 0·4789 |
| *RSV-B* | 430 (28·14) |  | 400 (28·49) | 12 (26·09) | 9 (26·47) | 9 (20·45) | 0·6793 |  | 272 (27·34) | 158 (29·64) | 0·3702 |
| *RSV-untyped* | 182 (19·34) |  | 156 (18·80) | 7 (21·21) | 6 (17·65) | 13 (29·55) | 0·3572 |  | 121 (20·54) | 61 (17·33) | 0·2617 |
| HRV | 882 (6·78) |  | 655 (9·82) | 61 (6·16) | 83 (3·19) | 83 (3·02) | <0·0001 |  | 570 (7·10) | 312 (6·27) | 0·0743 |
| HPIV | 867 (6·66) |  | 673 (10·09) | 44 (4·44) | 72 (2·77) | 78 (2·84) | <0·0001 |  | 543 (6·76) | 324 (6·51) | 0·6060 |
| *HPIV-1* | 205 (13·28) |  | 168 (12·64) | 10 (13·51) | 15 (23·08) | 12 (15·79) | 0·0975 |  | 140 (14·18) | 65 (11·67) | 0·1867 |
| *HPIV-2* | 165 (10·69) |  | 97 (7·30) | 7 (9·46) | 31 (47·69) | 30 (39·47) | <0·0001 |  | 97 (9·83) | 68 (12·21) | 0·1713 |
| *HPIV-3* | 479 (31·02) |  | 414 (31·15) | 22 (29·73) | 17 (26·15) | 26 (34·21) | 0·7652 |  | 308 (31·21) | 171 (30·70) | 0·8816 |
| *HPIV-4* | 68 (8·13) |  | 46 (7·07) | 5 (11·36) | 8 (12·31) | 9 (11·84) | 0·1592 |  | 35 (6·70) | 33 (10·51) | 0·0690 |
| *HPIV-untyped* | 12 (1·74) |  | 10 (1·98) | 2 (4·65) | 0 (0·00) | 0 (0·00) | 0·2077 |  | 7 (1·65) | 5 (1·88) | >0·9999 |
| HAdV | 498 (3·83) |  | 356 (5·34) | 59 (5·96) | 52 (2·00) | 31 (1·13) | <0·0001 |  | 311 (3·87) | 187 (3·76) | 0·7788 |
| HCoV | 326 (2·51) |  | 195 (2·92) | 24 (2·42) | 50 (1·92) | 57 (2·08) | 0·0148 |  | 211 (2·63) | 115 (2·31) | 0·2885 |
| HBoV | 320 (2·46) |  | 287 (4·30) | 16 (1·62) | 9 (0·35) | 8 (0·29) | <0·0001 |  | 215 (2·68) | 105 (2·11) | 0·0490 |
| HMPV | 299 (2·30) |  | 227 (3·40) | 15 (1·52) | 25 (0·96) | 32 (1·17) | <0·0001 |  | 176 (2·19) | 123 (2·47) | 0·3276 |
| Bacteria | 1 402/5 178  (27·08) |  | 544/1 583  (34·37) | 65/184  (35·33) | 342/1 447  (23·64) | 451/1 964  (22·96) | <0·0001 |  | 953/3 323  (28·68) | 449/1 855  (24·20) | 0·0006 |
| *S. pneumoniae* | 388 (7·49) |  | 228 (14·40) | 13 (7·07) | 79 (5·46) | 68 (3·46) | <0·0001 |  | 261 (7·85) | 127 (6·85) | 0·2055 |
| *K. pneumoniae* | 360 (6·95) |  | 58 (3·66) | 6 (3·26) | 118 (8·15) | 178 (9·06) | <0·0001 |  | 266 (8·00) | 94 (5·07) | <0·0001 |
| *P. aeruginosa* | 301 (5·81) |  | 19 (1·20) | 5 (2·72) | 93 (6·43) | 184 (9·37) | <0·0001 |  | 194 (5·84) | 107 (5·77) | 0·9672 |
| *M. pneumoniae* | 248 (4·79) |  | 166 (10·49) | 38 (20·65) | 30 (2·07) | 14 (0·71) | <0·0001 |  | 147 (4·42) | 101 (5·44) | 0·1137 |
| *H. influenzae* | 174 (3·36) |  | 85 (5·37) | 10 (5·43) | 39 (2·70) | 40 (2·04) | <0·0001 |  | 122 (3·67) | 52 (2·80) | 0·1137 |
| *S. aureus* | 169 (3·26) |  | 60 (3·79) | 8 (4·35) | 52 (3·59) | 49 (2·49) | 0·1000 |  | 129 (3·88) | 40 (2·16) | 0·0011 |

**Supplementary table 1 (Continued)**

|  | **Case type** | | | **ICU admission** | | | **Clinical outcome** | | | **Season of infection** | | |
| --- | --- | --- | --- | --- | --- | --- | --- | --- | --- | --- | --- | --- |
|  | **Inpatient** | **Outpatient** | **P value** | **Yes** | **No** | **P value** | **Discharge** | **Death** | **P value** | **Cold** | **Warm** | **P value** |
| Virus | 4 030/10 553 (38·19) | 736/2 456 (29·97) | <0·0001 | 571/1 870 (30·53) | 4 115/11 059 (37·21) | <0·0001 | 3 817/11 383 (33·53) | 37/121 (30·58) | 0·5565 | 2 881/7 356 (39·17) | 1 885/5 653 (33·35) | <0·0001 |
| IFV | 1 104 (10·46) | 293 (11·93) | 0·0374 | 163 (8·72) | 1 154 (10·43) | 0·0257 | 1 079 (9·48) | 19 (15·70) | 0·0306 | 928 (12·62) | 469 (8·30) | <0·0001 |
| *IFV-A* | 574 (35·09) | 193 (67·72) | <0·0001 | 82 (19·66) | 685 (45·55) | <0·0001 | 581 (58·39) | 14 (82·35) | 0·0816 | 536 (43·12) | 231 (34·07) | 0·0001 |
| *IFV-B* | 287 (17·54) | 74 (25·96) | 0·0011 | 24 (5·76) | 337 (22·41) | <0·0001 | 330 (33·17) | 2 (11·76) | 0·1089 | 231 (18·58) | 130 (19·17) | 0·7986 |
| *IFV-C* | 13 (1·70) | 1 (0·35) | 0·1291 | 1 (1·04) | 13 (1·36) | >0·999 | 12 (1·21) | 0 (0·00) | >0·9999 | 10 (1·31) | 4 (1·38) | >0·9999 |
| *IFV-untyped* | 81 (10·57) | 18 (6·32) | 0·0474 | 11 (11·46) | 88 (9·21) | 0·5933 | 90 (9·05) | 2 (11·76) | 0·6623 | 70 (9·19) | 29 (10·03) | 0·7626 |
| RSV | 1 219 (11·55) | 147 (5·99) | <0·0001 | 155 (8·29) | 1 211 (10·95) | 0·0006 | 1 015 (8·92) | 4 (3·31) | 0·0455 | 953 (12·96) | 413 (7·31) | <0·0001 |
| *RSV-A* | 579 (40·72) | 46 (43·40) | 0·6608 | 84 (52·50) | 541 (39·55) | 0·0022 | 441 (50·29) | 2 (50·00) | >0·9999 | 450 (45·82) | 175 (32·05) | <0·0001 |
| *RSV-B* | 405 (28·48) | 25 (23·58) | 0·3323 | 69 (43·12) | 361 (26·39) | <0·0001 | 298 (33·98) | 1 (25·00) | >0·9999 | 268 (27·29) | 162 (29·67) | 0·3515 |
| *RSV-untyped* | 147 (17·60) | 35 (33·02) | 0·0003 | 4 (5·56) | 178 (20·48) | 0·0034 | 155 (17·67) | 1 (25·00) | 0·5421 | 137 (21·14) | 45 (15·36) | 0·0465 |
| HRV | 793 (7·51) | 89 (3·62) | <0·0001 | 124 (6·63) | 747 (6·75) | 0·8828 | 656 (5·76) | 8 (6·61) | 0·8398 | 461 (6·27) | 421 (7·45) | 0·0088 |
| HPIV | 784 (7·43) | 83 (3·38) | <0·0001 | 89 (4·76) | 761 (6·88) | 0·0007 | 685 (6·02) | 5 (4·13) | 0·4988 | 423 (5·75) | 444 (7·85) | <0·0001 |
| *HPIV-1* | 187 (12·76) | 18 (23·08) | 0·0144 | 25 (6·58) | 180 (15·46) | <0·0001 | 137 (20·63) | 0 (0·00) | 0·5890 | 111 (13·59) | 94 (12·93) | 0·7609 |
| *HPIV-2* | 154 (10·50) | 11 (14·10) | 0·4156 | 15 (3·95) | 150 (12·89) | <0·0001 | 149 (22·44) | 2 (40·00) | 0·3159 | 108 (13·22) | 57 (7·84) | 0·0009 |
| *HPIV-3* | 444 (30·29) | 35 (44·87) | 0·0097 | 61 (16·05) | 418 (35·91) | <0·0001 | 354 (53·31) | 3 (60·00) | >0·9999 | 204 (24·97) | 275 (37·83) | <0·0001 |
| *HPIV-4* | 57 (7·52) | 11 (14·10) | 0·0707 | 10 (10·64) | 58 (7·82) | 0·4577 | 56 (8·43) | 0 (0·00) | >0·9999 | 30 (7·23) | 38 (9·03) | 0·4100 |
| *HPIV-untyped* | 8 (1·31) | 4 (5·13) | 0·0371 | 0 (0·00) | 12 (1·90) | 0·6127 | 11 (1·66) | 0 (0·00) | >0·9999 | 6 (1·78) | 6 (1·70) | >0·9999 |
| HAdV | 418 (3·96) | 80 (3·26) | 0·1145 | 80 (4·28) | 414 (3·74) | 0·2937 | 389 (3·42) | 3 (2·48) | 0·8007 | 255 (3·47) | 243 (4·30) | 0·0161 |
| HCoV | 284 (2·69) | 42 (1·71) | 0·0063 | 37 (1·98) | 285 (2·58) | 0·1455 | 290 (2·55) | 4 (3·31) | 0·5542 | 173 (2·35) | 153 (2·71) | 0·2200 |
| HBoV | 293 (2·78) | 27 (1·10) | <0·0001 | 37 (1·98) | 265 (2·40) | 0·3063 | 219 (1·92) | 0 (0·00) | 0·1777 | 143 (1·94) | 177 (3·13) | <0·0001 |
| HMPV | 240 (2·27) | 59 (2·40) | 0·7591 | 31 (1·66) | 263 (2·38) | 0·0645 | 249 (2·19) | 1 (0·83) | 0·5258 | 188 (2·56) | 111 (1·96) | 0·0296 |
| Bacteria | 1 312/4 753 (27·60) | 90/425 (21·18) | 0·0051 | 445/1 055 (42·18) | 948/4 088 (23·19) | <0·0001 | 935/4 067 (22·99) | 27/62 (43·55) | 0·0003 | 612/2 314 (26·45) | 790/2 864 (27·58) | 0·3771 |
| *S. pneumoniae* | 354 (7·45) | 34 (8·00) | 0·7505 | 112 (10·62) | 270 (6·6) | <0·0001 | 205 (5·04) | 6 (9·68) | 0·1326 | 187 (8·08) | 201 (7·02) | 0·1641 |
| *K. pneumoniae* | 334 (7·03) | 26 (6·12) | 0·5440 | 125 (11·85) | 234 (5·72) | <0·0001 | 304 (7·47) | 11 (17·74) | 0·0064 | 138 (5·96) | 222 (7·75) | 0·0139 |
| *P. aeruginosa* | 288 (6·06) | 13 (3·06) | 0·0153 | 108 (10·24) | 193 (4·72) | <0·0001 | 269 (6·61) | 9 (14·52) | 0·0344 | 109 (4·71) | 192 (6·70) | 0·0028 |
| *M. pneumoniae* | 231 (4·86) | 17 (4·00) | 0·4984 | 79 (7·49) | 167 (4·09) | <0·0001 | 88 (2·16) | 1 (1·61) | >0·9999 | 106 (4·58) | 142 (4·96) | 0·5710 |
| *H. influenzae* | 158 (3·32) | 16 (3·76) | 0·7321 | 59 (5·59) | 113 (2·76) | <0·0001 | 108 (2·66) | 1 (1·61) | >0·9999 | 98 (4·24) | 76 (2·65) | 0·0022 |
| *S. aureus* | 157 (3·30) | 12 (2·82) | 0·6960 | 59 (5·59) | 110 (2·69) | <0·0001 | 127 (3·12) | 6 (9·68) | 0·0140 | 79 (3·41) | 90 (3·14) | 0·6397 |

Data are n (%). Chi-square test or Fisher’s exact test were used for age group, gender, case type, ICU admission, clinical outcome, or season of infection. Positive rate was calculated by taking the positive number of each pathogen as the numerator and the total number of cases that underwent the tests for each specific pathogen as denominator. Virus: positive rate of any virus; Bacteria: positive rate of any bacteria. The viral positive rate of 13 009 CAP patients who had all the eight viral pathogens tested; the bacterial positive rate of 5 178 CAP patients who had all the six bacterial pathogens tested.

**Supplementary table 2.** **Positive rate of respiratory pathogens among patients with SCAP and non-SCAP by age group, gender, case type, and season of infection in Chinese mainland, 2009‒2020.**

|  | **Total** | |  | **Age group** | | | | | | | |
| --- | --- | --- | --- | --- | --- | --- | --- | --- | --- | --- | --- |
|  |  |  |  | **Children (≤ 5 years)** | | **Adolescents (18‒60 years)** | | **Adults (18‒60 years)** | | **The elderly (>60 years)** | |
|  | **Non-SCAP** | **SCAP** |  | **Non-SCAP** | **SCAP** | **Non-SCAP** | **SCAP** | **Non-SCAP** | **SCAP** | **Non-SCAP** | **SCAP** |
| Virus | 3 834/10 314  (37·17) | 932/2 695  (34·58) * |  | 2 829/5 690  (49·72) | 561/981  (57·19) ** | 282/878  (32·12) | 40/112  (35·71) | 405/1 969  (20·57) | 124/633  (19·59) | 318/1 777  (17·90) | 207/969  (21·36) * |
| IFV | 1 126 (10·92) | 271 (10·06) |  | 683 (12·00) | 97 (9·89) | 95 (10·82) | 15 (13·39) | 207 (10·51) | 53 (8·37) | 141 (7·93) | 106 (10·94) ** |
| *IFV-A* | 610 (39·33) | 157 (42·43) |  | 342 (30·08) | 49 (22·37) * | 38 (36·54) | 5 (38·46) | 129 (71·67) | 31 (75·61) | 101 (77·69) | 72 (74·23) |
| *IFV-B* | 313 (20·18) | 48 (12·97) ** |  | 227 (19·96) | 20 (9·13) | 32 (30·77) | 3 (23·08) | 31 (17·22) | 7 (17·07) | 23 (17·69) | 18 (18·56) |
| *IFV-C* | 7 (0·83) | 7 (3·35) * |  | 2 (0·44) | 4 (6·90) ** | 1 (1·37) | 0 (0·00) | 3 (1·67) | 1 (2·44) | 1 (0·77) | 2 (2·06) |
| *IFV-untyped* | 74 (8·79) | 25 (11·96) |  | 43 (9·37) | 11 (18·97) * | 5 (6·85) | 5 (38·46) ** | 19 (10·56) | 2 (4·88) | 7 (5·38) | 7 (7·22) |
| RSV | 1 121 (10·87) | 245 (9·09) ** |  | 1 013 (17·80) | 209 (21·30) ** | 47 (5·35) | 7 (6·25) | 27 (1·37) | 10 (1·58) | 34 (1·91) | 19 (1·96) |
| *RSV-A* | 500 (39·75) | 125 (46·30) * |  | 453 (38·75) | 110 (46·81) * | 18 (46·15) | 2 (28·57) | 16 (66·67) | 3 (30·00) | 13 (50·00) | 10 (55·56) |
| *RSV-B* | 353 (28·06) | 77 (28·52) |  | 333 (28·49) | 67 (28·51) | 8 (20·51) | 4 (57·14) | 5 (20·83) | 4 (40·00) | 7 (26·92) | 2 (11·11) |
| *RSV-untyped* | 149 (19·40) | 33 (19·08) |  | 133 (19·22) | 23 (16·67) | 6 (23·08) | 1 (14·29) | 3 (12·50) | 3 (30·00) | 7 (26·92) | 6 (33·33) |
| HRV | 675 (6·54) | 207 (7·68) * |  | 522 (9·17) | 133 (13·56) ** | 46 (5·24) | 15 (13·39) ** | 58 (2·95) | 25 (3·95) | 49 (2·76) | 34 (3·51) |
| HPIV | 732 (7·10) | 135 (5·01) ** |  | 582 (10·23) | 91 (9·28) | 40 (4·56) | 4 (3·57) | 57 (2·89) | 15 (2·37) | 53 (2·98) | 25 (2·58) |
| *HPIV-1* | 175 (13·69) | 30 (11·28) |  | 146 (13·20) | 22 (9·87) | 10 (14·29) | 0 (0·00) | 12 (23·53) | 3 (21·43) | 7 (13·73) | 5 (20·00) |
| *HPIV-2* | 144 (11·27) | 21 (7·89) |  | 90 (8·14) | 7 (3·14) ** | 6 (8·57) | 1 (25·00) | 24 (47·06) | 7 (50·00) | 24 (47·06) | 6 (24·00) |
| *HPIV-3* | 397 (31·06) | 82 (30·83) |  | 353 (31·92) | 61 (27·35) | 19 (27·14) | 3 (75·00) | 12 (23·53) | 5 (35·71) | 13 (25·49) | 13 (52·00) * |
| *HPIV-4* | 59 (8·43) | 9 (6·62) |  | 42 (7·53) | 4 (4·30) | 5 (12·50) | 0 (0·00) | 5 (9·80) | 3 (21·43) | 7 (13·73) | 2 (8·00) |
| *HPIV-untyped* | 12 (2·05) | 0 (0·00) |  | 10 (2·26) | 0 (0·00) | 2 (5·13) | 0 (0·00) | 0 (0·00) | 0 (0·00) | 0 (0·00) | 0 (0·00) |
| HAdV | 400 (3·88) | 98 (3·64) |  | 290 (5·10) | 66 (6·73) * | 56 (6·38) | 3 (2·68) | 38 (1·93) | 14 (2·21) | 16 (0·90) | 15 (1·55) |
| HBoV | 248 (2·40) | 72 (2·67) |  | 224 (3·94) | 63 (6·42) | 14 (1·59) | 2 (1·79) | 6 (0·30) | 3 (0·47) | 4 (0·23) | 4 (0·41) |
| HcoV | 262 (2·54) | 64 (2·37) |  | 176 (3·09) | 19 (1·94) * | 18 (2·05) | 6 (5·36) * | 37 (1·88) | 13 (2·05) | 31 (1·74) | 26 (2·68) |
| HMPV | 251 (2·43) | 48 (1·78) * |  | 193 (3·39) | 34 (3·47) | 14 (1·59) | 1 (0·89) | 22 (1·12) | 3 (0·47) | 22 (1·24) | 10 (1·03) |
| Bacteria | 902/3 773  (23·91) | 500/1 405  (35·59) ** |  | 406/1 242  (32·69) | 138/341  (40·47) ** | 54/155  (34·84) | 11/29  (37·93) | 203/1 030  (19·71) | 139/417  (33·33) ** | 239/1 346  (17·76) | 212/618  (34·30) ** |
| *K. pneumoniae* | 195 (5·17) | 165 (11·74) ** |  | 32 (2·58) | 26 (7·62) ** | 5 (3·23) | 1 (3·45) | 69 (6·70) | 49 (11·75) ** | 89 (6·61) | 89 (14·40) ** |
| *P. aeruginosa* | 155 (4·11) | 146 (10·39) ** |  | 11 (0·89) | 8 (2·35) * | 3 (1·94) | 2 (6·90) | 52 (5·05) | 41 (9·83) | 89 (6·61) | 95 (15·37) ** |
| *S. pneumoniae* | 276 (7·32) | 112 (7·97) |  | 185 (14·90) | 43 (12·61) | 10 (6·45) | 3 (10·34) | 45 (4·37) | 34 (8·15) ** | 36 (2·67) | 32 (5·18) ** |
| *S. aureus* | 89 (2·36) | 80 (5·69) ** |  | 34 (2·74) | 26 (7·62) ** | 6 (3·87) | 2 (6·90) | 24 (2·33) | 28 (6·71) ** | 25 (1·86) | 24 (3·88) ** |
| *H. influenzae* | 122 (3·23) | 52 (3·70) |  | 64 (5·15) | 21 (6·16) | 10 (6·45) | 0 (0·00) | 25 (2·43) | 14 (3·36) | 23 (1·71) | 17 (2·75) |
| *M. pneumoniae* | 202 (5·35) | 46 (3·27) ** |  | 135 (10·87) | 31 (9·09) | 34 (21·94) | 4 (13·79) | 25 (2·43) | 5 (1·20) | 8 (0·59) | 6 (0·97) |

**Supplementary table 2 (Continued)**

|  | **Sex** | | | | **Case type** | | | | **Season of infection** | | | |
| --- | --- | --- | --- | --- | --- | --- | --- | --- | --- | --- | --- | --- |
|  | **Male** | | **Female** | | **Inpatients** | | **Outpatients** | | **Cold** | | **Warm** | |
|  | **Non-SCAP** | **SCAP** | **Non-SCAP** | **SCAP** | **Non-SCAP** | **SCAP** | **Non-SCAP** | **SCAP** | **Non-SCAP** | **SCAP** | **Non-SCAP** | **SCAP** |
| Virus | 2356/6 258 (37·65) | 628/1 775  (35·38) | 1 478/4 056 (36·44) | 304/920  (33·04) | 3 141/8 000  (39·26) | 889/2 553  (34·82) ** | 693/2 314  (29·95) | 43/142  (30·28) | 2 300/5 869  (39.19) | 581/1 487  (39.07) | 1 534/4 445  (34.51) | 351/1 208  (29.06) |
| IFV | 694 (11·09) | 186 (10·48) | 432 (10·65) | 85 (9·24) | 851 (10·64) | 253 (9·91) | 275 (11·88) | 18 (12·68) | 718 (12.23) | 210 (14.12) | 408 (9.18) | 61 (5.05) ** |
| *IFV-A* | 373 (39·30) | 108 (40·91) | 237 (39·37) | 49 (46·23) | 429 (33·44) | 145 (41·08) ** | 181 (67·54) | 12 (70·59) | 408 (41.80) | 128 (47.94) | 202 (35.13) | 29 (28.16) |
| *IFV-B* | 180 (18·97) | 35 (13·26) * | 133 (22·09) | 13 (12·26) * | 241 (18·78) | 46 (13·03) * | 72 (26·87) | 2 (11·76) | 197 (20.18) | 34 (12.73) ** | 116 (20.17) | 14 (13.59) |
| *IFV-C* | 5 (1·02) | 5 (3·33) | 2 (0·57) | 2 (3·39) | 7 (1·22) | 6 (3·12) | 0 (0·00) | 1 (5·88) | 6 (1.01) | 4 (2.38) | 1 (0.40) | 3 (7.32) ** |
| *IFV-untyped* | 44 (8·98) | 22 (14·67) * | 30 (8·52) | 3 (5·08) | 58 (10·10) | 23 (11·98) | 16 (5·97) | 2 (11·76) | 51 (8.59) | 19 (11.31) | 23 (9.27) | 6 (14.63) |
| RSV | 703 (11·23) | 170 (9·58) * | 418 (10·31) | 75 (8·15) * | 980 (12·25) | 239 (9·36) ** | 141 (6·09) | 6 (4·23) | 771 (13.14) | 182 (12.24) | 350 (7.87) | 63 (5.22) ** |
| *RSV-A* | 307 (38·23) | 93 (48·44) ** | 193 (42·42) | 32 (41·03) | 456 (39·38) | 123 (46·59) * | 44 (44·00) | 2 (33·33) | 350 (44.47) | 100 (51.28) | 150 (31.85) | 25 (33.33) |
| *RSV-B* | 222 (27·65) | 50 (26·04) | 131 (28·79) | 27 (34·62) | 331 (28·58) | 74 (28·03) | 22 (22·00) | 3 (50·00) | 213 (27.06) | 55 (28.21) | 140 (29.72) | 22 (29.33) |
| *RSV-untyped* | 98 (20·85) | 23 (19·33) | 51 (17·11) | 10 (18·52) | 115 (17·22) | 32 (19·16) | 34 (34·00) | 1 (16·67) | 117 (22.46) | 20 (15.75) | 32 (12.96) | 13 (28.26) ** |
| HRV | 424 (6·78) | 146 (8·23) * | 251 (6·19) | 61 (6·63) | 593 (7·41) | 200 (7·83) | 82 (3·54) | 7 (4·93) | 348 (5.93) | 113 (7.60) * | 327 (7.36) | 94 (7.78) |
| HPIV | 456 (7·29) | 87 (4·90) ** | 276 (6·80) | 48 (5·22) | 654 (8·18) | 130 (5·09) ** | 78 (3·37) | 5 (3·52) | 355 (6.05) | 68 (4.57) * | 377 (8.48) | 67 (5.55) |
| *HPIV-1* | 125 (15·51) | 15 (8·29) * | 50 (10·59) | 15 (17·65) | 158 (13·11) | 29 (11·11) | 17 (23·29) | 1 (20·00) | 89 (13.38) | 22 (14.47) | 86 (14.03) | 8 (7.02) * |
| *HPIV-2* | 85 (10·55) | 12 (6·63) | 59 (12·50) | 9 (10·59) | 134 (11·12) | 20 (7·66) | 10 (13·70) | 1 (20·00) | 93 (13.98) | 15 (9.87) | 51 (8.32) | 6 (5.26) |
| *HPIV-3* | 251 (31·14) | 57 (31·49) | 146 (30·93) | 25 (29·41) | 365 (30·29) | 79 (30·27) | 32 (43·84) | 3 (60·00) | 170 (25.56) | 34 (22.37) | 227 (37.03) | 48 (42.11) |
| *HPIV-4* | 29 (6·68) | 6 (6·82) | 30 (11·28) | 3 (6·25) | 48 (7·66) | 9 (6·87) | 11 (15·07) | 0 (0·00) | 24 (7.00) | 6 (8.33) | 35 (9.80) | 3 (4.69) |
| *HPIV-untyped* | 7 (1·97) | 0 (0·00) | 5 (2·18) | 0 (0·00) | 8 (1·57) | 0 (0·00) | 4 (5·48) | 0 (0·00) | 6 (2.11) | 0 (0.00) | 6 (2.01) | 0 (0.00) |
| HAdV | 238 (3·80) | 73 (4·11) | 162 (3·99) | 25 (2·72) | 325 (4·06) | 93 (3·64) | 75 (3·24) | 5 (3·52) | 212 (3.61) | 43 (2.89) | 188 (4.23) | 55 (4.55) |
| HBoV | 169 (2·70) | 46 (2·59) | 79 (1·95) | 26 (2·83) | 226 (2·83) | 67 (2·62) | 22 (0·95) | 5 (3·52) * | 112 (1.91) | 31 (2.08) | 136 (3.06) | 41 (3.39) |
| HCoV | 170 (2·72) | 41 (2·31) | 92 (2·27) | 23 (2·50) | 225 (2·81) | 59 (2·31) | 37 (1·60) | 5 (3·52) | 138 (2.35) | 35 (2.35) | 124 (2.79) | 29 (2.40) |
| HMPV | 148 (2·36) | 28 (1·58) * | 103 (2·54) | 20 (2·17) | 193 (2·41) | 47 (1·84) | 58 (2·51) | 1 (0·70) | 160 (2.73) | 28 (1.88) | 91 (2.05) | 20 (1.66) |
| Bacteria | 604/2 371 (25·47) | 349/952 (36·66) ** | 298/1 402 (21·26) | 151/453 (33·33) ** | 822/3 388 (24·26) | 490/1 365 (35·90) ** | 80/385 (20·78) | 10/40 (25·00) | 366/1 643 (22.28) | 246/671 (36.66) ** | 536/2 130 (25.16) | 254/734 (34.60) ** |
| *K. pneumoniae* | 144 (6·07) | 122 (12·82) ** | 51 (3·64) | 43 (9·49) ** | 169 (4·99) | 165 (12·09) ** | 26 (6·75) | 0 (0·00) | 70 (4.26) | 68 (10.13) ** | 125 (5.87) | 97 (13.22) ** |
| *P. aeruginosa* | 93 (3·92) | 101 (10·61) ** | 62 (4·42) | 45 (9·93) ** | 146 (4·31) | 142 (10·40) ** | 9 (2·34) | 4  (10·00) * | 44 (2.68) | 65 (9.69) ** | 111 (5.21) | 81 (11.04) ** |
| *S. pneumoniae* | 193 (8·14) | 68 (7·14) | 83 (5·92) | 44 (9·71) ** | 244 (7·20) | 110 (8·06) | 32 (8·31) | 2 (5·00) | 132 (8.03) | 55 (8.20) | 144 (6.76) | 57 (7.77) |
| *S. aureus* | 64 (2·70) | 65 (6·83) ** | 25 (1·78) | 15 (3·31) | 80 (2·36) | 77 (5·64) ** | 9 (2·34) | 3 (7·50) | 38 (2.31) | 41 (6.11) ** | 51 (2.39) | 39 (5.31) ** |
| *H. influenzae* | 85 (3·58) | 37 (3·89) | 37 (2·64) | 15 (3·31) | 106 (3·13) | 52 (3·81) | 16 (4·16) | 0 (0·00) | 67 (4.08) | 31 (4.62) | 55 (2.58) | 21 (2.86) |
| *M. pneumoniae* | 119 (5·02) | 28 (2·94) ** | 83 (5·92) | 18 (3·97) | 186 (5·49) | 45 (3·30) ** | 16 (4·16) | 1 (2·50) | 78 (4.75) | 28 (4.17) | 124 (5.82) | 18 (2.45) |

Data are n (%). Chi-square test or Fisher’s exact test were used for comparisons among different groups. ^*^, p<0·05; ^**^, p<0·01. Virus: positive rate of any virus; Bacteria: positive rate of any bacteria. Positive rate was calculated by taking the positive number of each pathogen as the numerator and the total number of cases that underwent the tests for each specific pathogen as denominator. The viral positive rate of 2 695 SCAP patients who had all the eight viral pathogens tested; the bacterial positive rate of 1 405 SCAP patients who had all the six bacterial pathogens tested

**Supplementary table 3. Clinical features among CAP patients infected with different respiratory pathogens.**

|  | IFV | RSV | HPIV | HAdV | HMPV | HCoV | HBoV | HRV | *P. aeruginosa* | *K. pneumoniae* | *S. aureus* | *S. pneumoniae* | *H. influenzae* | *M. pneumoniae* |
| --- | --- | --- | --- | --- | --- | --- | --- | --- | --- | --- | --- | --- | --- | --- |
| Symptoms | n=1690 | n=1508 | n=884 | n=554 | n=307 | n=336 | n=310 | n=880 | n=310 | n=374 | n=171 | n=432 | n=195 | n=411 |
| Fever | 1382  (81.78) | 1084  (71.88) | 682  (77.15) | 482  (87.00) | 263  (85.67) | 271  (80.65) | 216  (69.68) | 599  (68.07) | 174  (56.13) | 215  (57.49) | 87  (50.88) | 276  (63.89) | 131  (67.18) | 339  (82.48) |
| Cough | 1332  (78.82) | 1323  (87.73) | 761  (86.09) | 438  (79.06) | 261  (85.02) | 279  (83.04) | 274  (88.39) | 779  (88.52) | 202  (65.16) | 245  (65.51) | 116  (67.84) | 376  (87.04) | 173  (88.72) | 381  (92.70) |
| Rhinorrhea | 428  (25.33) | 349  (23.14) | 147  (16.63) | 118  (21.30) | 86  (28.01) | 47  (13.99) | 42  (13.55) | 156  (17.73) | 10  (3.23) | 23  (6.15) | 14  (8.19) | 70  (16.20) | 29  (14.87) | 58  (14.11) |
| Sore throat | 364  (21.54) | 122  (8.09) | 75  (8.48) | 90  (16.25) | 29  (9.45) | 37  (11.01) | 20  (6.45) | 69  (7.84) | 7  (2.26) | 26  (6.95) | 6  (3.51) | 22  (5.09) | 18  (9.23) | 40  (9.73) |
| Expectoration | 660  (39.05) | 620  (41.11) | 374  (42.31) | 183  (33.03) | 122  (39.74) | 138  (41.07) | 122  (39.35) | 424  (48.18) | 176  (56.77) | 197  (52.67) | 79  (46.20) | 282  (65.28) | 129  (66.15) | 215  (52.31) |
| Chest pain | 69  (4.08) | 24  (1.59) | 13  (1.47) | 19  (3.43) | 11  (3.58) | 15  (4.46) | 10  (3.23) | 28  (3.18) | 14  (4.52) | 22  (5.88) | 9  (5.26) | 36  (8.33) | 17  (8.72) | 14  (3.41) |
| Tachypnea | 168  (9.94) | 215  (14.26) | 112  (12.67) | 70  (12.64) | 32  (10.42) | 23  (6.85) | 62  (20.00) | 146  (16.59) | 52  (16.77) | 74  (19.79) | 43  (25.15) | 90  (20.83) | 46  (23.59) | 74  (18.00) |
| Dyspneic | 202  (11.95) | 257  (17.04) | 159  (17.99) | 64  (11.55) | 36  (11.73) | 57  (16.96) | 49  (15.81) | 152  (17.27) | 101  (32.58) | 97  (25.94) | 44  (25.73) | 105  (24.31) | 51  (26.15) | 35  (8.52) |
| Headache | 222  (13.14) | 56  (3.71) | 31  (3.51) | 64  (11.55) | 17  (5.54) | 15  (4.46) | 10  (3.23) | 22  (2.50) | 12  (3.87) | 21  (5.61) | 12  (7.02) | 16  (3.70) | 9  (4.62) | 20  (4.87) |
| Fatigue | 187  (11.07) | 50  (3.32) | 19  (2.15) | 38  (6.86) | 17  (5.54) | 14  (4.17) | 5  (1.61) | 43  (4.89) | 29  (9.35) | 54  (14.44) | 15  (8.77) | 26  (6.02) | 23  (11.79) | 23  (5.60) |
| Bellyache | 10  (0.59) | 9  (0.60) | 6  (0.68) | 6  (1.08) | 4  (1.30) | 2  (0.60) | 2  (0.65) | 8  (0.91) | 6  (1.94) | 8  (2.14) | 1  (0.58) | 6  (1.39) | 2  (1.03) | 7  (1.70) |
| Diarrhea | 54  (3.20) | 127  (8.42) | 63  (7.13) | 29  (5.23) | 11  (3.58) | 13  (3.87) | 22  (7.10) | 60  (6.82) | 5  (1.61) | 13  (3.48) | 13  (7.60) | 27  (6.25) | 21  (10.77) | 21  (5.11) |
| Signs |  |  |  |  |  |  |  |  |  |  |  |  |  |  |
| Pulmonary rales | 351  (20.77) | 522  (34.62) | 240  (27.15) | 124  (22.38) | 84  (27.36) | 65  (19.35) | 105  (33.87) | 297  (33.75) | 99  (31.94) | 131  (35.03) | 48  (28.07) | 178  (41.20) | 95  (48.72) | 156  (37.96) |
| Laboratory findings | n=541 | n=593 | n=345 | n=170 | n=110 | n=103 | n=159 | n=370 | n=164 | n=216 | n=76 | n=239 | n=117 | n=229 |
| WBC, ×109/L [4-10] | |  |  |  |  |  |  |  |  |  |  |  |  |  |
| Increased | 168  (31.05) | 183  (30.86) | 107  (31.01) | 58  (34.12) | 31  (28.18) | 27  (26.21) | 74  (46.54) | 152  (41.08) | 88  (53.66) | 113  (52.31) | 39  (51.32) | 124  (51.88) | 59  (50.43) | 67  (29.26) |
| Decreased | 47  (8.69) | 13  (2.19) | 13  (3.77) | 7  (4.12) | 3  (2.73) | 10  (9.71) | 6  (3.77) | 13  (3.51) | 14  (8.54) | 12  (5.56) | 2  (2.63) | 9  (3.77) | 3  (2.56) | 12  (5.24) |
| Lym, ×109/L [0·8-4] | |  |  |  |  |  |  |  |  |  |  |  |  |  |
| Increased | 227  (41.96) | 370  (62.39) | 186  (53.91) | 68  (40.00) | 60  (54.55) | 42  (40.78) | 80  (50.31) | 177  (47.84) | 21  (12.80) | 36  (16.67) | 35  (46.05) | 118  (49.37) | 42  (35.90) | 83  (36.24) |
| Decreased | 156  (28.84) | 82  (13.83) | 51  (14.78) | 32  (18.82) | 16  (14.55) | 28  (27.18) | 30  (18.87) | 73  (19.73) | 106  (64.63) | 131  (60.65) | 29  (38.16) | 49  (20.50) | 38  (32.48) | 49  (21.40) |
| Neu, % [5-70] |  |  |  |  |  |  |  |  |  |  |  |  |  |  |
| Increased | 167  (30.87) | 79  (13.32) | 53  (15.36) | 34  (20.00) | 12  (10.91) | 25  (24.27) | 33  (20.75) | 82  (22.16) | 108  (65.85) | 134  (62.04) | 31  (40.79) | 59  (24.69) | 44  (37.61) | 49  (21.40) |
| Decreased | 215  (39.74) | 364  (61.38) | 186  (53.91) | 68  (40.00) | 60  (54.55) | 47  (45.63) | 79  (49.69) | 177  (47.84) | 18  (10.98) | 37  (17.13) | 33  (43.42) | 104  (43.51) | 36  (30.77) | 79  (34.50) |

Data are n (%). WBC: white blood cell count; Lym: lymphocyte; Neu: neutrophilic granulocyte percentage.

**Supplementary table 3. Co-infection rate of CAP patients by age.**

|  | **Co-infection** |  | **Viral-bacterial** |  | **Viral-viral** |  | **Bacterial-bacterial** |
| --- | --- | --- | --- | --- | --- | --- | --- |
| Total (n=3 552) | 798 (22·47) |  | 405 (11·40) |  | 264 (7·43) |  | 129 (3·63) |
| Age group, n (%) |  |  |  |  |  |  |  |
| Children (≤5 years) (n=1 440) | 572 (39·72) |  | 317 (22·01) |  | 227 (15·76) |  | 28 (1·94) |
| Adolescents (6**‒**17 years) (n=145) | 30 (20·69) |  | 20 (13·79) |  | 6 (4·14) |  | 4 (2·76) |
| Adults (18**‒**60 years) (n=898) | 78 (8·69) |  | 22 (2·45) |  | 13 (1·45) |  | 43 (4·79) |
| The elderly (>60 years) (n=1 069) | 118 (11·04) |  | 46 (4·30) |  | 18 (1·68) |  | 54 (5·05) |
| P value | <0·0001 |  | <0·0001 |  | <0·0001 |  | <0·0001 |
| Season of infection, n (%) |  |  |  |  |  |  |  |
| Cold (n=1 780) | 434 (24·38) |  | 223 (12·53) |  | 152 (8·54) |  | 59 (3·31) |
| Warm (n=1 772) | 364 (20·54) |  | 182 (10·27) |  | 112 (6·32) |  | 70 (3·95) |
| P value | 0·0069 |  | 0·0391 |  | 0·0140 |  | 0·3560 |

Data are n (%), Chi square test or Fisher’s exact test were used for comparisons for age groups or season group. The co-infection rate of 3 552 CAP patients who had all the 14 respiratory pathogens tested.

**Supplementary table 4. Co-infection rate compared between SCAP and non-SCAP patients by age.**

|  | **Co-infection** | | |  | **Viral-bacterial** | | |  | **Bacterial-bacterial** | | |  | **Viral-viral** | | |
| --- | --- | --- | --- | --- | --- | --- | --- | --- | --- | --- | --- | --- | --- | --- | --- |
|  | **Non-SCAP** | **SCAP** | **p value** |  | **Non-SCAP** | **SCAP** | **p value** |  | **Non-SCAP** | **SCAP** | **p value** |  | **Non-SCAP** | **SCAP** | **p value** |
| Total | 550/2 506  (21·95) | 248/1 046  (23·71) | 0·2701 |  | 276  (11·01) | 129  (12·33) | 0·2848 |  | 67  (2·67) | 62  (5·93) | <0·0001 |  | 207  (8·26) | 57  (5·45) | 0·0045 |
| Age group, n (%) |  |  |  |  |  |  |  |  |  |  |  |  |  |  |  |
| Children (≤5 years) | 432/1 124  (38·43) | 140/316  (44·30) | 0·0689 |  | 226  (20·11) | 91  (28·80) | 0·0013 |  | 21  (1·87) | 7  (2·22) | 0·8698 |  | 185  (16·46) | 42  (13·29) | 0·2013 |
| Adolescents (6**‒**17 years) | 25/125  (20·00) | 5/20  (25·00) | 0·5648 |  | 16  (12·80) | 4  (20·00) | 0·4815 |  | 4  (3·20) | 0  (0·00) | >0·9999 |  | 5  (4·00) | 1  (5·00) | >0·9999 |
| Adults (18**‒**60 years) | 42/592  (7·09) | 36/306  (11·76) | 0·0257 |  | 14  (2·36) | 8  (2·61) | 0·9988 |  | 21  (3·55) | 22  (7·19) | 0·024 |  | 7  (1·18) | 6  (1·96) | 0·3838 |
| The elderly (>60 years) | 51/665  (7·67) | 67/404  (16·58) | <0·0001 |  | 20  (3·01) | 26  (6·44) | 0·0116 |  | 21  (3·16) | 33  (8·17) | <0·0001 |  | 10  (1·50) | 8  (1·98) | 0·7324 |
| Season of infection, n (%) |  |  |  |  |  |  |  |  |  |  |  |  |  |  |  |
| Cold | 286/1 243  (23·01) | 148/537  (27·56) | 0·0463 |  | 131  (10·54) | 92  (17·13) | <0·0001 |  | 34  (2·74) | 25  (4·66) | 0·0533 |  | 121  (9·73) | 31  (5·77) | 0·0080 |
| Warm | 264/1 263  (20·90) | 100/509  (19·65) | 0·5980 |  | 145  (11·48) | 37  (7·27) | 0·0106 |  | 33  (2·61) | 37  (7·27) | <0·0001 |  | 86  (6·81) | 26  (5·11) | 0·2211 |

Data are n (%), Chi square test or Fisher’s exact test were used for comparisons for non-SCAP and SCAP groups

**Supplementary table 5. Coinfection patterns for different age groups between SCAP and non-SCAP patients.** Only the top 5 pairs of the coinfection viruses were listed.

|  | **Co-infection rate (%)** |  | **Co-infection rate (%)** |  | **Co-infection rate (%)** |
| --- | --- | --- | --- | --- | --- |
| Children (≤ 5 years) | | | | | |
| Total (n=1 440) | | SCAP (n=316) | | non-SCAP (n=1 124) | |
| RSV-HRV | 91 (6·32) | RSV-HRV | 19 (6·01) | RSV-HRV | 72 (6·41) |
| RSV-*S. pneumoniae* | 57 (3·96) | RSV-*S. pneumoniae* | 16 (5·06) | RSV-IFV | 42 (3·74) |
| RSV-IFV | 53 (3·68) | RSV-HPIV | 12 (3·80) | RSV-*S. pneumoniae* | 41 (3·65) |
| RSV-HPIV | 49 (3·40) | RSV-*S. aureus* | 12 (3·80) | RSV-HPIV | 37 (3·29) |
| HRV-HPIV | 43 (2·99) | RSV-IFV | 11 (3·48) | HRV-HPIV | 34 (3·02) |
| Adolescents (6‒17 years) | | | | | |
| Total (n=145) | | SCAP (n=20) | | non-SCAP (n=125) | |
| *S. pneumoniae*-*H. influenzae* | 5  (3.45) | RSV-HRV | 1  (5·00) | *S. pneumoniae*-*H. influenzae* | 5  (4·00) |
| HRV-*M. pneumoniae* | 5  (3.45) | HPIV-*M. pneumoniae* | 1  (5·00) | HRV-*M. pneumoniae* | 5  (4·00) |
| HRV-*S. pneumoniae* | 3  (2.07) | RSV-IFV | 1  (5·00) | HRV-*S. pneumoniae* | 3  (2·40) |
| HRV-*H. influenzae* | 3  (2.07) | HRV-IFV | 1  (5·00) | HRV-*H. influenzae* | 3  (2·40) |
| *S. pneumoniae*-*M. pneumoniae* | 3  (2.07) | *S. pneumoniae*-IFV | 1  (5·00) | *S. pneumoniae*-*M. pneumonia*e | 3  (2·40) |
| Adults (18‒60 years) | | | | | |
| Total (n=898) | | SCAP (n=306) | | non-SCAP (n=592) | |
| *S. pneumoniae*-*H. influenzae* | 17  (1.89) | *K. pneumoniae*-*P. aeruginosa* | 8  (2·61) | *S. pneumoniae*-*H. influenzae* | 16  (2·70) |
| *K. pneumoniae*-*P. aeruginosa* | 11  (1.22) | *S. pneumoniae*-*S. aureus* | 5  (1·63) | HRV-*S. pneumonia*e | 3  (0·51) |
| *S. pneumoniae*-*S. aureus* | 7  (0.78) | *S. aureus*-*P. aeruginosa* | 4  (1·31) | HRV-*H. influenzae* | 3  (0·51) |
| *S. pneumoniae*-*K. pneumoniae* | 6  (0.67) | *S. pneumoniae*-*K. pneumoniae* | 3  (0·98) | HRV-HAdV | 3  (0·51) |
| *K. pneumoniae*-*S. aureus* | 6  (0.67) | *H. influenzae*-*K. pneumoniae* | 3  (0·98) | *S. pneumoniae*-*K. pneumoniae* | 3  (0·51) |
| The elderly (>60 years) | | | | | |
| Total (1 069) | | SCAP (n=404) | | non-SCAP (n=665) | |
| *K. pneumoniae*-*P. aeruginosa* | 34  (3·18) | *K. pneumoniae*-*P. aeruginosa* | 24  (5·94) | *K. pneumoniae*-*P. aeruginosa* | 10  (1·50) |
| IFV-*P. aeruginosa* | 11  (1·03) | IFV-*P. aeruginosa* | 8  (1·98) | *K. pneumoniae*-*S. aureus* | 5  (0·75) |
| *S. aureus*-*P. aeruginosa* | 10  (0·94) | *S. aureus*-*P. aeruginosa* | 8  (1·98) | HRV-*S. pneumoniae* | 4  (0·60) |
| *K. pneumoniae*-*S. aureus* | 9  (0·84) | *S. pneumoniae*-*K. pneumonia*e | 5  (1·24) | HRV-HPIV | 3  (0·45) |
| *S. pneumoniae*-*H. influenzae* | 7  (0·65) | *H. influenzae*-*P. aeruginosa* | 5  (1·24) | *S. pneumoniae*-*H. influenzae* | 3  (0·45) |

**Supplementary table 6.** **The association between SCAP and age, sex, season of infection, pathogen prevalence.**

|  | **SCAP (%)** | **Univariate analysis** | |  | **Multivariate analysis** | |
| --- | --- | --- | --- | --- | --- | --- |
|  |  | **Crude OR (95% CI)** | **P  value** |  | **Adjusted OR (95% CI)** | **P  value** |
| Juveniles <18 years (n=1175) |  |  |  |  |  |  |
| Sex |  |  |  |  |  |  |
| Female | 72 (16·55) | Reference |  |  | Reference |  |
| Male | 173 (23·38) | 1·54 (1·13–2·09) | 0·0056 |  | **1·55 (1·12–2·12)** | **0·0073** |
| Season of infection |  |  |  |  |  |  |
| Warm | 93 (18·24) | Reference |  |  | Reference |  |
| Cold | 152 (22·86) | 1·33 (1·00–1·77) | 0·0537 |  | **1·37 (1·00–1·86)** | **0·0481** |
| Pathogen prevalence |  |  |  |  |  |  |
| All negative | 61 (17·73) | Reference |  |  | Reference |  |
| RSV-S. aureus | 10 (62·50) | 7·73 (2·71–22·08) | 0·0001 |  | **7·07 (2·45–20·40)** | **0·0003** |
| HAdV | 16 (51·61) | 4·95 (2·32–10·55) | <0·0001 |  | **4·86 (2·27–10·44)** | **<0·0001** |
| *K. pneumoniae* | 5 (41·67) | 3·31 (1·02–10·79) | 0·0467 |  | **3·54 (1·07–11·69)** | **0·0383** |
| RSV-*H. influenzae* | 8 (40·00) | 3·09 (1·21–7·89) | 0·0181 |  | **2·62 (1·02–6·74)** | **0·0464** |
| HBoV | 9 (28·12) | 1·82 (0·80–4·12) | 0·1535 |  | 2·04 (0·89–4·66) | 0·0914 |
| HRV | 28 (29·47) | 1·94 (1·15–3·26) | 0·0127 |  | **1·91 (1·13–3·22)** | **0·0161** |
| RSV-*S. pneumoniae* | 11 (25·58) | 1·59 (0·76–3·34) | 0·2156 |  | 1·52 (0·72–3·21) | 0·2666 |
| RSV-HPIV | 9 (22·50) | 1·35 (0·61–2·97) | 0·4611 |  | 1·26 (0·57–2·79) | 0·5750 |
| *S. aureus* | 4 (22·22) | 1·33 (0·42–4·17) | 0·6296 |  | 1·22 (0·38–3·85) | 0·7391 |
| RSV-HRV | 20 (21·51) | 1·27 (0·72–2·24) | 0·4069 |  | 1·16 (0·66–2·06) | 0·6028 |
| RSV | 28 (19·86) | 1·15 (0·70–1·89) | 0·5831 |  | 1·01 (0·61–1·68) | 0·9705 |
| *S. pneumoniae* | 10 (15·62) | 0·86 (0·41–1·78) | 0·6832 |  | 0·83 (0·40–1·72) | 0·6162 |
| *M. pneumoniae* | 9 (13·43) | 0·72 (0·34–1·53) | 0·3934 |  | 0·80 (0·37–1·71) | 0·5641 |
| *H. influenzae* | 3 (13·64) | 0·73 (0·21–2·55) | 0·6252 |  | 0·69 (0·20–2·42) | 0·5636 |
| HPIV | 7 (11·11) | 0·58 (0·25–1·33) | 0·1998 |  | 0·54 (0·23–1·25) | 0·1500 |
| IFV | 7 (9·46) | 0·48 (0·21–1·11) | 0·0858 |  | 0·47 (0·21–1·08) | 0·0766 |
| Adults ≥18 years (n=1819) |  |  |  |  |  |  |
| Sex |  |  |  |  |  |  |
| Female | 215 (34·62) | Reference |  |  |  |  |
| Male | 422 (35·23) | 1·03 (0·84–1·26) | 0·7980 |  |  |  |
| Season of infection |  |  |  |  |  |  |
| Warm | 341 (34·69) | Reference |  |  |  |  |
| Cold | 296 (35·41) | 1·03 (0·85–1·25) | 0·7494 |  |  |  |
| Pathogen prevalence |  |  |  |  |  |  |
| All negative | 364 (30·74) | Reference |  |  | Reference |  |
| *P. aeruginosa*-*K. pneumoniae* | 32 (72·73) | 6·01 (3·06–11·80) | <0·0001 |  | **6·01 (3·06–11·80)** | **<0·0001** |
| *S. pneumoniae* | 37 (50·00) | 2·25 (1·40–3·61) | 0·0007 |  | **2·25 (1·40–3·61)** | **0·0007** |
| *P. aeruginosa* | 51 (49·04) | 2·17 (1·45–3·25) | 0·0002 |  | **2·17 (1·45–3·25)** | **0·0002** |
| *K. pneumoniae* | 46 (47·42) | 2·03 (1·34–3·08) | 0·0009 |  | **2·03 (1·34–3·08)** | **0·0009** |
| HRV | 12 (44·44) | 1·80 (0·84–3·89) | 0·1333 |  | 1·80 (0·84–3·89) | 0·1333 |
| *S. aureus* | 14 (40·00) | 1·50 (0·76–2·99) | 0·2462 |  | 1·50 (0·76–2·99) | 0·2462 |
| *H. influenzae* | 13 (38·24) | 1·39 (0·69–2·82) | 0·3535 |  | 1·39 (0·69–2·82) | 0·3535 |
| IFV | 36 (37·89) | 1·37 (0·89–2·12) | 0·1494 |  | 1·37 (0·89–2·12) | 0·1494 |
| HCoV | 8 (36·36) | 1·29 (0·54–3·10) | 0·5727 |  | 1·29 (0·54–3·10) | 0·5727 |
| *M. pneumoniae* | 7 (25·00) | 0·75 (0·32–1·78) | 0·5159 |  | 0·75 (0·32–1·78) | 0·5159 |
| HPIV | 9 (24·32) | 0·72 (0·34–1·55) | 0·4058 |  | 0·72 (0·34–1·55) | 0·4058 |
| HMPV | 3 (21·43) | 0·61 (0·17–2·22) | 0·4566 |  | 0·61 (0·17–2·22) | 0·4566 |
| *S. pneumoniae*-*H. influenzae* | 5 (20·83) | 0·59 (0·22–1·60) | 0·3020 |  | 0·59 (0·22–1·60) | 0·3020 |

Bold font indicates P value <0·05. Results are not shown when the number of positive pathogens is less than 10 in juveniles <18 years or adults ≥18 years.

**Supplementary table 7.** **The association between ICU admission of patients and age, sex, season of infection, pathogen prevalence for adults≥18 years.**

|  | **ICU admission rate (%)** |  | **Univariate analysis** | |  | **Multivariate analysis** | |
| --- | --- | --- | --- | --- | --- | --- | --- |
|  |  |  | **Crude OR**  **(95% CI)** | **P value** |  | **Adjusted OR**  **(95% CI)** | **P value** |
| Age group |  |  |  |  |  |  |  |
| Adults (18‒60 years) | 227 (28.52) |  | Reference |  |  |  |  |
| The elderly (>60 years) | 242 (25.83) |  | 0.87  (0.71–1.08) | 0·2091 |  |  |  |
| Sex |  |  |  |  |  |  |  |
| Female | 148 (25.26) |  | Reference |  |  |  |  |
| Male | 321 (27.99) |  | 1.15  (0.92–1.44) | 0·2264 |  | - |  |
| Season of infection |  |  |  |  |  |  |  |
| Warm | 264 (27.85) |  | Reference |  |  |  |  |
| Cold | 205 (26.11) |  | 0.92  (0.74–1.13) | 0·4189 |  | - |  |
| Pathogen prevalence |  |  |  |  |  |  |  |
| All negative | 263 (22.21) |  | Reference |  |  | Reference |  |
| *P. aeruginosa*-*K. pneumoniae* | 25 (56.82) |  | 4.61  (2.50–8.50) | <0·0001 |  | **4.61**  **(2.50–8.50)** | **<0·0001** |
| *S. aureus* | 18 (51.43) |  | 3.71  (1.88–7.30) | 0·0001 |  | **3.71**  **(1.88–7.30)** | **0·0001** |
| *K. pneumoniae* | 41 (42.27) |  | 2.56  (1.68–3.92) | <0·001 |  | **2.56**  **(1.68–3.92)** | **<0·001** |
| *P. aeruginosa* | 40 (38.46) |  | 2.19  (1.44–3.32) | 0·0002 |  | **2.19**  **(1.44–3.32)** | **0·0002** |
| *S. pneumoniae* | 27 (36.49) |  | 2.01  (1.23–3.29) | 0·0054 |  | **2.01**  **(1.23–3.29)** | **0·0054** |
| HMPV | 5 (35.71) |  | 1.95  (0.65–5.86) | 0·2364 |  | 1.95  (0.65–5.86) | 0·2364 |
| *H. influenzae* | 11 (32.35) |  | 1.67  (0.81–3.48) | 0·1670 |  | 1.67  (0.81–3.48) | 0·1670 |
| IFV | 28 (29.47) |  | 1.46  (0.92–2.32) | 0·1061 |  | 1.46  (0.92–2.32) | 0·1061 |
| *M. pneumoniae* | 6 (21.43) |  | 0.96  (0.38–2.38) | 0·9214 |  | 0.96  (0.38–2.38) | 0·9214 |
| *S. pneumoniae-H. influenzae* | 5 (20.83) |  | 0.92  (0.34–2.49) | 0·8721 |  | 0.92  (0.34–2.49) | 0·8721 |

Bold font indicates P value <0·05.

**Supplementary table 8. The association between death of patients and pathogen prevalence by conditional logistic regression in adults ≥18 years.**

|  | **Mortality (%)** | **OR (95% CI)** | **P value** |
| --- | --- | --- | --- |
| Pathogen prevalence |  |  |  |
| All negative | 23 (12.85) |  |  |
| *P. aeruginosa*-*K. pneumoniae* | 4 (57.14) | **11.54 (1.94–68.61)** | **0·0072** |
| *S. pneumoniae* | 4 (36.36) | **7.55 (1.54–37.01)** | **0·0127** |
| IFV | 5 (38.46) | **3.38 (1.05–10.82)** | **0·0405** |
| *K. pneumoniae* | 4 (19.05) | 1.79 (0.51–6.29) | 0·3648 |
| *M. pneumoniae* | 1 (20.00) | 1.32 (0.13–12.88) | 0·8133 |
| HRV | 1 (20.00) | 1.16 (0.13–10.67) | 0·8959 |
| *P. aeruginosa* | 2 (12.50) | 1.13 (0.23–5.57) | 0·8807 |
| *S. aureus* | 1 (14.29) | 1.09 (0.11–10.73) | 0·9384 |

Because there were only three deaths among juveniles<18 years, they were not included in the analysis.

The sample size was insufficient, but we think the clinical significance is also important.

Variables of age, sex, season of infection were used as match variable.

Bold font indicates P value <0·05.

**Supplementary figure** **1. Prevalence of pathogens in mono infection and coinfection determined in patients with CAP in the Chinese mainland, 2009‒2020.** Viral-mono: viral mono-infection, viral-viral: viral-viral coinfection, viral-bact: viral-bacterial coinfection, bact-bact: bacterial-bacterial coinfection, bact-mono: bacterial mono-infection, negative: no pathogen was positive for all 14 viral and bacterial pathogens tested. (A) Positive proportion of viruses, viral-viral coinfections, viral-bacteria coinfections, bacterial-bacterial coinfections, and bacteria in different age groups. (B) Heatmap of the co-infection rate of respiratory pathogens. The grid color represents the co-infection of respiratory pathogens among patients with CAP. Darker color of the grid indicate higher coinfection rates between the pair of pathogens.


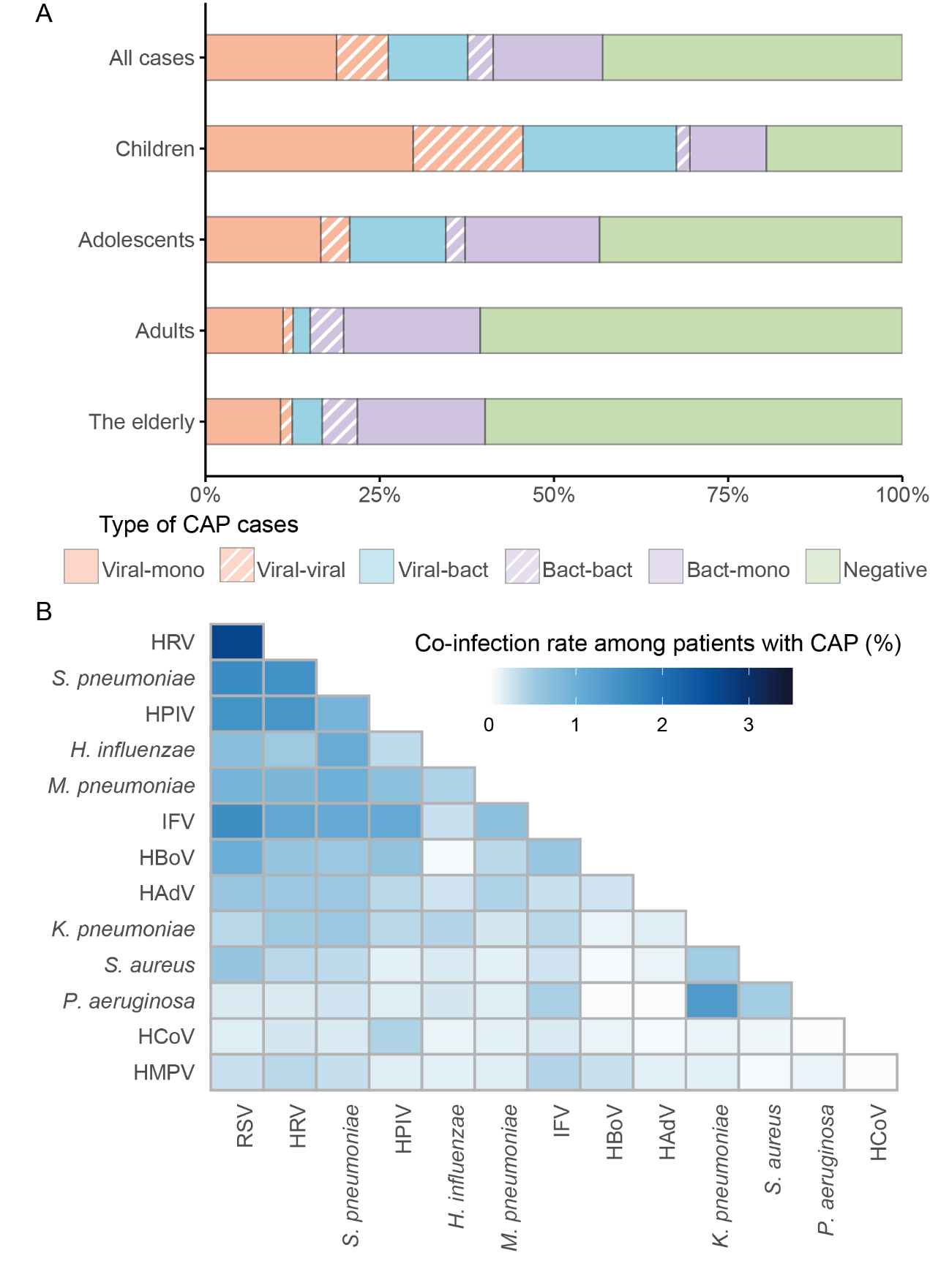


**Supplementary figure** **2. Comparison of coinfections rate between cold and warm season.** The grid color represents the co-infection rate of respiratory pathogens among patients in the warm season, The dot color represents the co-infection rate of respiratory pathogens among patients in the cold season. Bigger size and darker color of the circles indicate higher coinfection rates between the pair of pathogens.


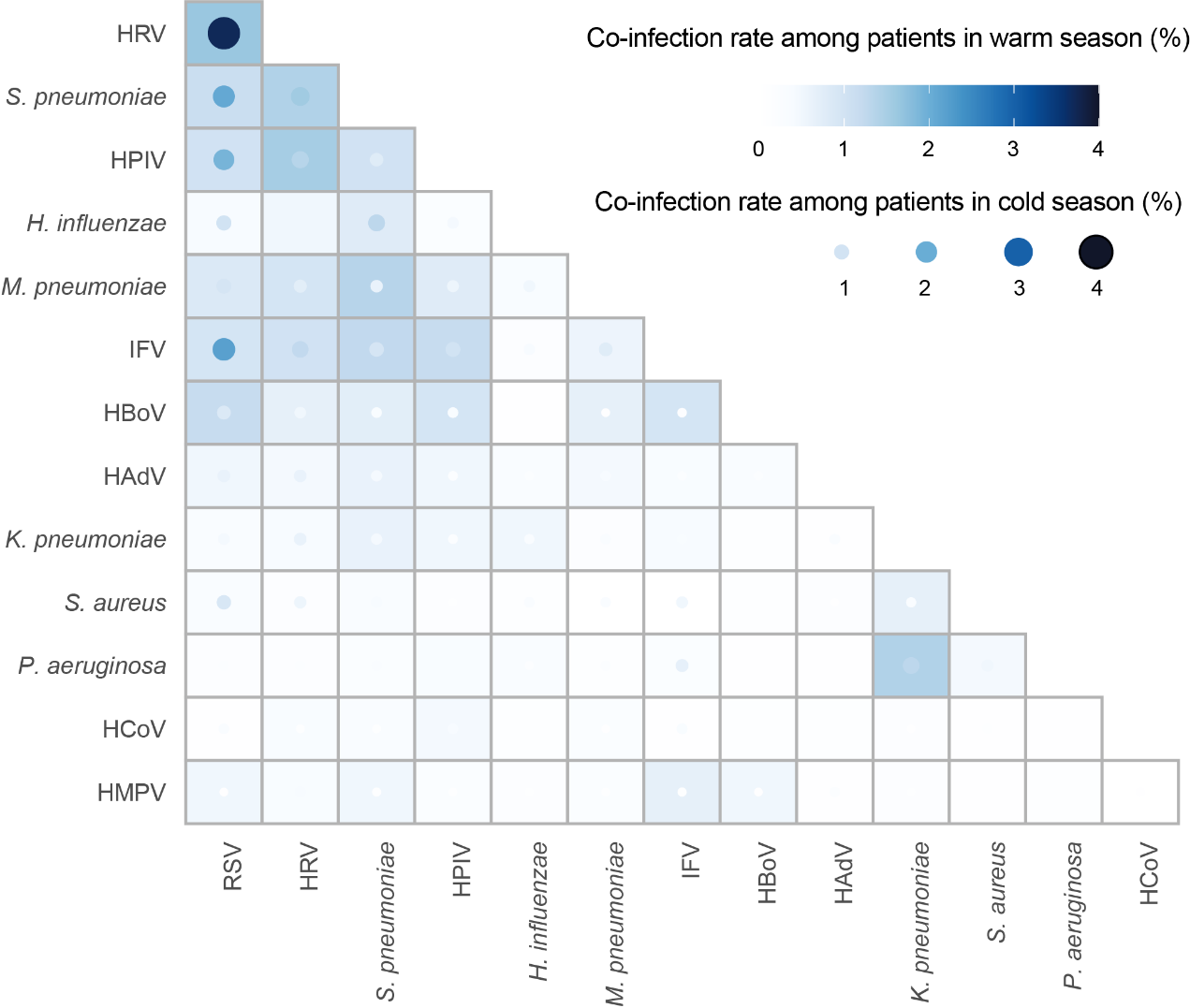


**Supplementary figure** **3. Comparison of coinfections rate between pre-pandemic years (2009‒2019) and COVID-19 pandemic year (2020).** The grid color represents the co-infection rate of respiratory pathogens among patients in the COVID-19 pandemic year (2020), The dot color represents the co-infection rate of respiratory pathogens among patients in the pre-pandemic years (2009‒2019). Bigger size and darker color of the circles indicate higher coinfection rates between the pair of pathogens.


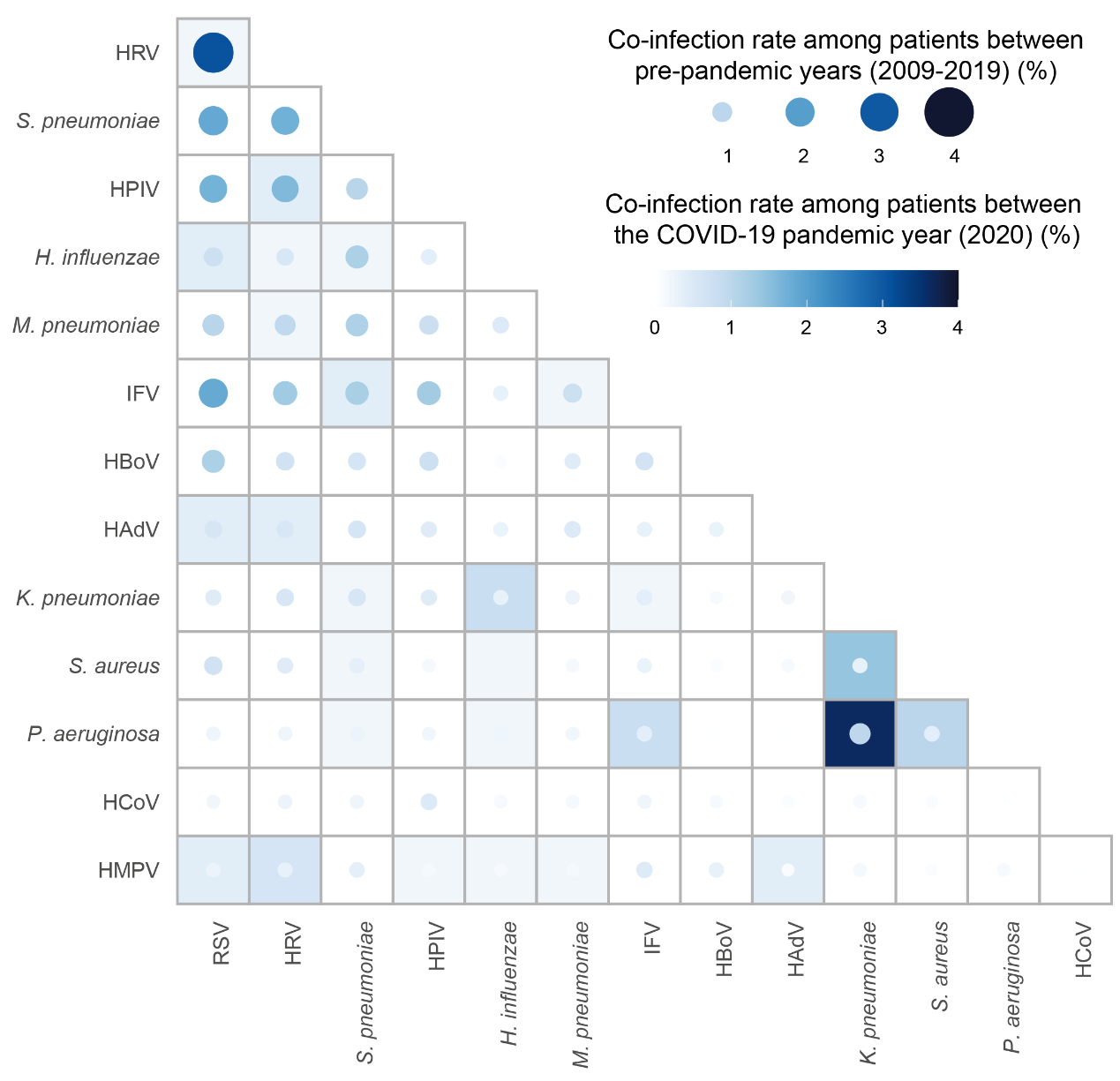


**Supplementary figure** **4. Comparison of the incidence of SCAP in different age groups in each season between pre-pandemic years (2009‒2019) and COVID-19 pandemic year (2020).** Blue and pink bar indicates the incidence of SCAP of different age groups in each season during the pre-pandemic years (2009‒2019) and during the first COVID-19 pandemic year (2020), respectively. Statistically significant changes were marked with asterisks (* p<0·05; ** p<0·01 based on chi-square test or Fisher’s exact test). (A) Comparison of the incidence of SCAP in different age groups in warm season between pre-pandemic years (2009‒2019) and COVID-19 pandemic year (2020). (B) Comparison of the incidence of SCAP in different age groups in cold season between pre-pandemic years (2009‒2019) and COVID-19 pandemic year (2020).


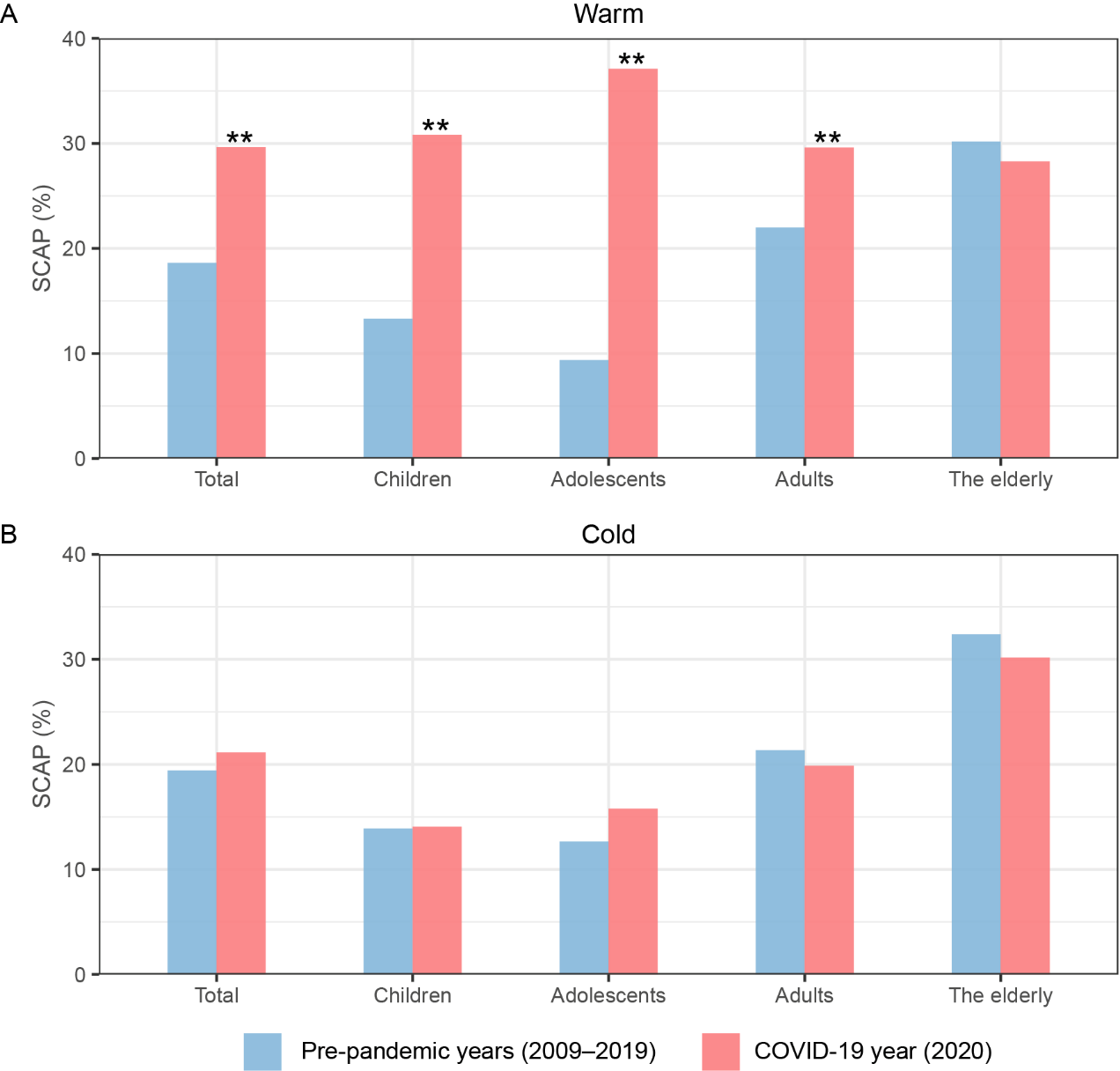


**The Chinese Centers for Disease Control and Prevention (CDC) Etiology of respiratory Surveillance Study Team**

1. Chinese Center for Disease Control and Prevention, Beijing, China: Wei-Zhong Yang; George F. Gao.

2. Division of Infectious Disease, Key Laboratory of Surveillance and Early-warning on Infectious Disease, Chinese Center for Disease Control and Prevention, Beijing, China: Zhong-Jie Li; Li-Ping Wang; Xiang Ren; Yi-Fei Wang; Sheng-Hong Lin; Cui-Hong Zhang; Meng-Jie Geng.

3. National Institute for Communicable Disease Control and Prevention, Chinese Center for Disease Control and Prevention, Beijing, China: Xin Wang; Huai-Qi Jing.

4. National Institute for Viral Disease Control and Prevention, Chinese Center for Disease Control and Prevention, Beijing, China: Wen-Bo Xu; Ai-Li Cui.

5. National Institute of Parasitic Diseases, Chinese Center for Disease Control and Prevention, Shanghai, China: Yu-Juan Shen; Yan-Yan Jiang.

6. Center of Disease Prevention and Control in Pudong New Area of Shanghai, Shanghai, China: Qiao Sun; Li-Peng Hao; Chu-Chu Ye.

7. State Key Laboratory of Pathogen and Biosecurity, Beijing Institute of Microbiology and Epidemiology, Beijing, China: Wei Liu; Xiao-Ai Zhang.

8. The Institute for Disease Prevention and Control of PLA, Beijing, China: Liu-Yu Huang; Yong Wang; Wen-Yi Zhang.

9. Wuhan University, Wuhan, China: Ying-Le Liu; Jian-Guo Wu; Qi Zhang.

10. Tongji Hospital, Tongji Medical College, Huazhong University of Science and

Technology, Wuhan, China: Wei-Yong Liu; Zi-Yong Sun.

11. Hubei Provincial Center for Disease Control and Prevention, Wuhan, China: Fa-Xian Zhan.

12. Jiangxi Provincial Center for Disease Control and Prevention, Nanchang, China: Ying Xiong.

13. Gansu Provincial Center for Disease Control and Prevention, Lanzhou, China: Lei Meng; De-Shan Yu.

14. Qinghai Provincial Center for Disease Control and Prevention, Xining, China: Chun-Xiang Wang; Sheng-Cang Zhao.

15. Inner Mongolia Autonomous Region Comprehensive Center for Disease Control and Prevention, Hohhot, China: Wen-Rui Wang; Xia Lei.

16. Lanzhou University, Lanzhou, China: Juan-Sheng Li.

17. Lanzhou Center for Disease Control and Prevention, Lanzhou, China: Yu-Hong Wang; Yan Zhang.

18. Baiyin Center for Disease Control and Prevention, Baiyin, China: Jun-Peng Yang; Yan-Bo Wang.

19. Tianshui Center for Disease Control and Prevention, Tianshui, China: Fu-Cai Quan; Zhi-Jun Xiong.

20. Wuwei Center for Disease Prevention and Control, Wuwei, China: Li-Ping Liang; Quan-E Chang.

21. Qingyang Center for Disease Control and Prevention, Qingyang, China: Yun Wang; Ping Wang.

22. Liaoning Provincial Center for Disease Control and Prevention, Shenyang, China: Zuo-Sen Yang; Ling-Ling Mao.

23. Tianjin Center for Disease Control and Prevention, Tianjin, China: Jia-Meng Li; Li-Kun Lv.

24. Heilongjiang Provincial Center for Disease Control and Prevention, Harbin, China: Jun Xu; Chang Shu.

25. Zhejiang University, Hangzhou, China: Xiao Chen; Yu Chen.

26. Zhejiang Center for Disease Control and Prevention, Hangzhou, China: Yan-Jun Zhang.

27. Jiangsu Provincial Center for Disease Control and Prevention, Nanjing, China: Lun-Biao Cui.

28. Fujian Center for Disease Control and Prevention, Fuzhou, China: Kui-Cheng Zheng.

29. Beilun People's Hospital, Ningbo, China: Xing-Guo Zhang.

30. Shanghai Municipal Center for Disease Control and Prevention, Shanghai, China: Xi Zhang; Li-Hong Tu.

31. Shanghai Public Health Clinical Center, Shanghai, China: Zhi-Gang Yi; Wei Wang.

32. Yunnan Center for Disease Control and Prevention, Kunming, China: Shi-Wen Zhao; Xiao-Fang Zhou.

33. Sichuan University, Chengdu, China: Xiao-Fang Pei; Tian-Li Zheng.

34. Chongqing Medical University, Chongqing, China: Xiao-Ni Zhong.

35. Chongqing Center for Disease Control and Prevention, Chongqing, China: Qin Li; Hua Ling.

36. Guizhou Center for Disease Control and Prevention, Guiyang, China: Ding-Ming Wang; Shi-Jun Li.

37. Sichuan Province Center for Disease Control and Prevention, Chengdu, China: Shu-Sen He.

38. Sun Yat-sen University, Guangzhou, China: Meng-Feng Li; Jun Li; Xun Zhu.

39. Guangdong Provincial Center for Disease Control and Prevention, Guangzhou, China: Chang-Wen Ke; Hong Xiao.

40. Guangzhou Municipal Center for Disease Control and Prevention, Guangzhou, China: Biao Di; Ying Zhang.

41. Zhujiang Hospital, Southern Medical University, Guangzhou, China: Hong-Wei Zhou; Nan Yu.

42. Jinan University, Guangzhou, China: Hong-Jian Li; Fang Yang.

43. The Third People's Hospital of Shenzhen, Shenzhen, China: Fu-Xiang Wang; Jun Wang.
